# Evidence-based Korean guidelines for the clinical management of multiple myeloma: addressing 12 key clinical questions

**DOI:** 10.1007/s44313-025-00055-9

**Published:** 2025-02-04

**Authors:** Sung-Hoon Jung, Youngil Koh, Min Kyoung Kim, Jin Seok Kim, Joon Ho Moon, Chang-Ki Min, Dok Hyun Yoon, Sung-Soo Yoon, Je-Jung Lee, Chae Moon Hong, Ka-Won Kang, Jihyun Kwon, Kyoung Ha Kim, Dae Sik Kim, Sung Yong Kim, Sung-Hyun Kim, Yu Ri Kim, Young Rok Do, Yeung-Chul Mun, Sung-Soo Park, Young Hoon Park, Ho Jin Shin, Hyeon-Seok Eom, Sang Eun Yoon, Sang Mee Hwang, Won Sik Lee, Myung-won Lee, Jun Ho Yi, Ji Yun Lee, Ji Hyun Lee, Ho Sup Lee, Sung-Nam Lim, Jihyang Lim, Ho-Young Yhim, Yoon Hwan Chang, Jae-Cheol Jo, Jinhyun Cho, Hyungwoo Cho, Yoon Seok Choi, Hee jeong Cho, Ari Ahn, Jong Han Choi, Hyun Jung Kim, Kihyun Kim

**Affiliations:** 1 Department of Hematology-Oncology, Chonnam National University Hwasun Hospital, Chonnam National University Medical School, Hwasun, Republic of Korea; 2 Division of Hematology-Oncology, Department of Internal Medicine, Seoul National University Hospital, Seoul National University College of Medicine, Seoul, Republic of Korea; 3 Division of Hemato-Oncology, Department of Internal Medicine, Yeungnam University College of Medicine, Daegu, Republic of Korea; 4 Division of Hematology, Department of Internal Medicine, Yonsei University College of Medicine, Severance Hospital, Seoul, Republic of Korea; 5 Department of Hematology/Oncology, Kyungpook National University Hospital, School of Medicine, Kyungpook National University, Daegu, Republic of Korea; 6 Seoul St. Mary’s Hospital, College of Medicine, The Catholic University of Korea, Seoul, Republic of Korea; 7 Department of Oncology, Asan Medical Center, University of Ulsan College of Medicine, Seoul, Republic of Korea; 8 Department of Nuclear Medicine, School of Medicine, Kyungpook National University, Daegu, Republic of Korea; 9 Division of Hematology-Oncology, Department of Internal Medicine, Korea University College of Medicine, Seoul, Republic of Korea; 10 Department of Internal Medicine, Hematology and Oncology, College of Medicine, Chungbuk National University, Cheongju, Republic of Korea; 11 Division of Hematology and Oncology, Department of Internal Medicine, Soonchunhyang University Seoul Hospital, Seoul, Republic of Korea; 12 Division of Oncology & Hematology, Department of Internal Medicine, Korea University Guro Hospital, Seoul, Republic of Korea; 13 Hematology & Oncology, Konkuk University Medical Center, Konkuk University School of Medicine, Seoul, Republic of Korea; 14 Division of Hematology-Oncology, Department of Internal Medicine, Dong-A University College of Medicine, Busan, Republic of Korea; 15 Division of Hematology-Oncology, Department of Internal Medicine, Dongsan Medical Center, Keimyung University School of Medicine, Daegu, Republic of Korea; 16 Department of Internal Medicine, Ewha Womans University College of Medicine, Seoul, Republic of Korea; 17 Division of Hematology-Oncology, Department of Internal Medicine, Pusan National University Hospital, Pusan National University School of Medicine, Busan, Republic of Korea; 18 Department of Hematology-Oncology, Center for Hematologic Malignancy, National Cancer Center, Goyang, Republic of Korea; 19 Division of Hematology-Oncology, Department of Medicine, Samsung Medical Center, Sungkyunkwan University School of Medicine, 81 Irwon-Ro, Gangnam-Gu, Seoul, Republic of Korea; 20 Department of Laboratory Medicine, Seoul National University Bundang Hospital, Seoul National University College of Medicine, Seongnam, Republic of Korea; 21 Department of Internal Medicine, Hematology, Inje University Busan Paik Hospital, Busan, Republic of Korea; 22 Division of Hematology and Oncology, Department of Internal Medicine, Chungnam National University Hospital, Daejeon, Republic of Korea; 23 Division of Hematology-Oncology, Department of Medicine, Chung-Ang University, Seoul, Republic of Korea; 24 Division of Hematology and Medical Oncology, Department of Internal Medicine, Seoul National University Bundang Hospital, Seoul National University College of Medicine, Seongnam, Republic of Korea; 25 Department of Internal Medicine, Kosin University College of Medicine, Kosin University Gospel Hospital, Busan, Republic of Korea; 26 Department of Internal Medicine, Inje University College of Medicine, Haeundae Paik Hospital, Busan, Republic of Korea; 27 Department of Laboratory Medicine, Eunpyeong St. Mary’s Hospital, College of Medicine, The Catholic University of Korea, Seoul, Republic of Korea; 28 Department of Internal Medicine, Jeonbuk National University Medical School, Jeonju, Republic of Korea; 29 Department of Laboratory Medicine, Seoul National University Hospital, Seoul National University College of Medicine, Seoul, Republic of Korea; 30 Department of Hematology and Oncology, Ulsan University Hospital, University of Ulsan College of Medicine, Ulsan, Republic of Korea; 31 Division of Hematology-Oncology, Department of Internal Medicine, Inha University Hospital, Inha University School of Medicine, Incheon, Republic of Korea; 32 Department of Laboratory Medicine, College of Medicine, Incheon St. Mary’s Hospital, The Catholic University of Korea, Seoul, Republic of Korea; 33 Department of Endocrine and Metabolism Medicine, Konkuk University Medical Center, Seoul, Republic of Korea; 34 Institute for Evidence-Based Medicine, Cochrane Korea College of Medicine, Korea University, Seoul, Republic of Korea

**Keywords:** Multiple myeloma, Guidelines, Meta-analysis, Treatment, Prognosis

## Abstract

Multiple myeloma (MM), a hematological malignancy, is characterized by malignant plasma cell proliferation in the bone marrow. Recent treatment advances have significantly improved patient outcomes associated with MM. In this study, we aimed to develop comprehensive, evidence-based guidelines for the diagnosis, prognosis, and treatment of MM. We identified 12 key clinical questions essential for MM management, guiding the extensive literature review and meta-analysis of the study. Our guidelines provide evidence-based recommendations by integrating patient preferences with survey data. These recommendations include current and emerging diagnostic tools, therapeutic agents, and treatment strategies. By prioritizing a patient-centered approach and rigorous data analysis, these guidelines were developed to enhance MM management, both in Korea and globally.

**Supplementary Information:**

The online version contains supplementary material available at 10.1007/s44313-025-00055-9.

## Introduction

Multiple myeloma (MM), is a hematologic malignancy with clonal malignant plasma cell proliferation within the bone marrow [1]. MM has been considered rare and incurable owing to the diagnostic challenges and low awareness. However, diagnostic technology advances and global population aging have led to a significant increase in the number of newly diagnosed MM cases. In 2021, approximately 2,018 new MM cases were reported in Korea and MM was the second most common hematological malignancy in the country [2].

Over the past two decades, MM treatment has improved substantially with the development of therapeutic agents, including proteasome inhibitors, immunomodulatory drugs, and monoclonal antibodies. Numerous clinical trials have evaluated the efficacy and safety of various treatment regimens, including two-, three-, and four-drug combinations incorporating these agents. Furthermore, imaging modalities have been increasingly used in MM diagnosis and prognosis at initial diagnosis, post-treatment, and relapse [3]. Furthermore, minimal residual disease (MRD) assessment via bone marrow examination has become a critical prognostic tool [4]. Based on these advancements and expert opinions, several guidelines have been proposed for MM diagnosis and treatment [5–7]. However, a systematic, evidence-based approach for MM guideline development is absent.

Therefore, in this study, we aimed to identify 12 key clinical questions pivotal to MM diagnosis, prognosis, and treatment to for novel guideline development based on an extensive literature review and meta-analysis. This approach differentiates our guideline from others owing to the focus on evidence-based recommendations along with expert opinion based only on literature review. Moreover, patient preferences, obtained through survey data, were incorporated into the guideline to ensure a patient-centered approach.

The recommendations presented in this guideline include both current therapeutic and diagnostic strategies as well as those anticipated to be introduced into the Korean healthcare system soon (Table 1). These evidence-based recommendations are based on rigorous data analysis and expert consensus. We hope that this guideline will inform clinical practice within Korea and provide valuable guidance to healthcare providers globally for MM management.

**Table 1:** Summary of recommendations

*KQ1*. Is a four-drug regimen more effective than a three-drug regimen for induction in patients with transplant-eligible MM?		
Recommendation	Grade	Level of evidence
For patients with newly diagnosed transplant-eligible MM, a four-drug regimen as initial therapy is recommended over a three-drug regimen owing to its better response rate	Should be considered (Do, conditional)	Moderate
*KQ2*. Is continuous therapy superior to fixed-duration first-line therapy in newly diagnosed patients with transplant-ineligible MM?		
Recommendation	Grade	Level of evidence
Maintaining first-line therapy is recommended for patients with newly diagnosed transplant-ineligible MM	Should be considered (Do, conditional)	Moderate
*KQ3*. Is initial or delayed ASCT more effective in patients with transplant-eligible MM?		
Recommendation	Grade	Level of evidence
Upfront ASCT is recommended rather than delayed transplantation in patients with transplant-eligible MM	Should be considered (Do, conditional)	Very low
*KQ4*. Is tandem transplantation better in improving OS as to a single ASCT?		
Recommendation	Grade	Level of evidence
Tandem ASCT generally does not lead to significant improvement in survival as compared to single ASCT and carries an increased risk of toxicity. Therefore, it is generally not recommended for patients with MM. However, it may be considered on a limited basis for high-risk MM	May be considered (Do not, conditional)	Low
*KQ5*. Does maintenance therapy after ASCT improve survival in patients with MM?		
Recommendation	Grade	Level of evidence
In patients with MM, maintenance therapy after ASCT significantly improves PFS and OS. Therefore, maintenance therapy is recommended	Should be considered (Do, conditional)	High
*KQ6*. Does treatment during biochemical relapse improve survival as compared to treatment during symptomatic relapse in relapsed MM?		
Recommendation	Grade	Level of evidence
In patients with relapsed MM, treatment at biochemical relapse is recommended over treatment at the onset of clinical symptoms	Should be considered (Do, conditional)	Low
*KQ7*. Is retreatment with previously effective agents a viable approach for relapsed MM?		
Recommendation	Grade	Level of evidence
When no other treatments are effective for patients with relapse, reusing a previously effective drug may be a viable option	Should be considered (Do, conditional)	Very low
*KQ8*. Is the use of antibiotics or antivirals effective for preventing infections during initial induction therapy in patients with newly diagnosed MM?		
Recommendation	Grade	Level of evidence
Prophylactic antibiotics are recommended during initial therapy in patients with newly diagnosed	Should be considered (Do, conditional)	Very low
Prophylactic antiviral agents are recommended during initial therapy in patients with newly diagnosed MM	Should be considered (Do, conditional)	Moderate
*KQ9.* Is the use of bone resorption inhibitors effective in MM treatment?		
Recommendation	Grade	Level of evidence
The use of pamidronate and zoledronate is recommended for the prevention and delay of SREs in patients with active MM	Is recommended (Do, strong)	Moderate
Denosumab can be recommended for the prevention and delay of SREs in patients with active MM, as it is not inferior to zoledronate	Should be considered (Do, conditional)	Low
*KQ10*. Are FDG PET/CT or MRI useful for predicting prognosis of patients with newly diagnosed MM?		
Recommendation	Grade	Level of evidence
In newly diagnosed MM, both FDG PET/CT and MRI have significant prognostic value; therefore, either imaging modality can be recommended for prognosis prediction	Should be considered (Do, conditional)	Very low
*KQ11*. Is the assessment of MRD useful in patients with newly diagnosed MM?		
Recommendation	Grade	Level of evidence
In newly diagnosed MM, the assessment of MRD is recommended	Should be considered (Do, conditional)	Very low
*KQ12*. Is immediate treatment initiation beneficial in patients with high-risk smoldering MM?		
Recommendation	Grade	Level of evidence
For high-risk asymptomatic MM, initiating treatment immediately is recommended to extend PFS and OS	Should be considered (Do, conditional)	Low

*KQ* key question, *MM* multiple myeloma, *ASCT* autologous stem cell transplantation, *PFS* progression-free survival, *OS* overall survival, *SREs* skeletal-related events, *FDG PET/CT* fluorodeoxyglucose positron emission tomography/computed tomography, *MRI* magnetic resonance imaging, *MRD* minimal residual disease

## Development of the Korean guideline

These guidelines were developed based on a systematic review following the Cochrane methodology recommendations [8]. The GRADE approach [9] was used to evaluate the quality of evidence and assign grades to recommendations.

### Identification of key questions

The development committee members were selected from the Korean Multiple Myeloma Working Party (KMMWP) under the Korean Society of Hematology. A survey was administered to committee members to formulate the key questions to be prioritized. The questions were prioritized based on the relevance of existing studies and the need for recommendations in current diagnostic and therapeutic practices.

### Literature search

The primary search terms for each key question were established by discussions between methodology experts and the respective committee members. A comprehensive search formula was designed and used for MEDLINE (PubMed) search. No limitations were imposed on publication year, language, or status. The identified articles were managed using EndNote with duplicate studies initially excluded based on the title, author, year, and journal, followed by a manual check to identify any duplicates. The MEDLINE, Embase, Cochrane, and KoreaMed databases were used in this study. Methodology experts and committee members conducted searches for each key question to minimize subjective judgments by individual members.

### Literature selection

Two committee members were assigned to review the literature for each key question to avoid duplication. The literature selection was conducted in accordance with the PRISMA Flow Diagram [10]. Inclusion and exclusion criteria were formulated for each key question based on the PICO framework (population, intervention, comparison, outcome). Owing to the limited high-quality evidence in this domain, no restrictions were imposed on the study design. Furthermore, studies without comparison groups were included to collect relevant information extensively.

### Assessing the quality of primary literature and data

The evaluation of evidence quality was performed in two stages. First, the quality of the individual primary studies was assessed followed by combining these assessments to evaluate overall level of evidence. Quality assessment of the primary studies and literature was conducted independently by members of the development committee for each key question. Any discrepancies in the evaluations were resolved by consensus. Second, evidence quality assessment was conducted based on the criteria established by the GRADE group (Tables 2 and 3). The level of evidence was then determined by deliberation among the methodology experts and the committee members responsible for each key question. Throughout this process, consistent criteria were imposed to ensure objectivity and guide clinical practice. The results of this appraisal are presented in the Supplementary material as a summary of findings (SoF) table. Each level of evidence was assigned based on the outcome measure with randomized studies used as reference when both randomized and nonrandomized studies were included for the same outcome. The level of evidence in the recommendations represents the most important primary outcome for each individual recommendation. Furthermore, the quality of evidence for studies addressing each key question was characterized to highlight the limitations and strengths of the available data.

**Table 2:** Level of evidence

Level of evidence	Definition
High	Evidence from well-conducted randomized clinical trials or meta-analyses with low risk of bias in study design and conduct, or from observational studies with no bias in study design or conduct and a very large effect size
Moderate	Evidence from randomized clinical trials or meta-analyses with some bias in study design and conduct or from observational studies with no bias in study design or conduct and a large effect size
Low	Evidence from randomized clinical trials or meta-analyses with significant bias in study design and conduct or from observational studies with no bias in study design and conduct
Very low	Evidence from observational studies or case reports with bias in study design and conduct or from poorly organized observational studies

**Table 3:** Definition of recommendation grade

Grade	Strength	Direction	Definitions
Is recommended	Strong	Do	When the benefits of a treatment or test clearly outweigh the risks, burdens, and costs
Should be considered	Conditional		When the benefits of a treatment or test likely outweigh the risks, burdens, and costs but are uncertain
May be considered	Conditional	Do not	When the risks, burdens, and costs of a treatment or test likely outweigh the benefits but are uncertain
Is not recommended	Strong		When the risks, burdens, and costs of a treatment or test clearly outweigh the benefits

### Summation of estimates (meta-analysis)

Meta-analyses were conducted when at least two results were available, except in cases with unexplained heterogeneity present among the included studies. If the study designs differed, the results were presented separately without aggregation. For single studies with multiple results, only one result was selected for consistent outcomes; while, for varying outcomes, all results were included. In cases with suspected duplication, the most recent publication or study with the largest sample size was included in the final meta-analysis. A random-effects model was applied to all meta-analyses. The *I*^2^ statistic was used to assess statistical heterogeneity. However, as statistical heterogeneity can be affected by the number of studies and events, clinical heterogeneity was also considered even in the absence of statistical heterogeneity. This meta-analysis incorporated all outcomes related to both the benefits and harms associated with the target intervention.

### Survey of patient preferences

For each key question, the development committee formulated survey questions corresponding to the research question. A survey was administered to 141 patients with MM undergoing treatment across 17 hospitals in South Korea.

## Key question 1. Is a four-drug regimen more effective than a three-drug regimen for induction in patients with transplant-eligible MM?

Overall, five prospective randomized studies were analyzed to compare the efficacy of three-(triplet) and four-drug regimens (quadruplet) as induction therapy in patients with transplant-eligible MM. Among these studies, only the CASSIOPEIA [11] and MYELOMA XI [12] trials provided data on progression-free survival (PFS) with sufficient follow-up for comparison. The other three studies, GMM-HD7, GRIFFIN, and the Phase II study, have not reported PFS outcomes yet, leading to comparisons based solely on response rates [13–15]. The CASSIOPEIA trial compared the triplet regimen VTD (bortezomib, thalidomide, dexamethasone) with the quadruplet regimen D-VTD (daratumumab, bortezomib, thalidomide, dexamethasone) with statistically significant improvement in PFS with the quadruplet regimen (HR, 0.47; 95% CI, 0.33–0.67; *p*< 0.0001) [11]. Similarly, the MYELOMA XI study compared the quadruplet regimen KRdc (carfilzomib, lenalidomide, dexamethasone, cyclophosphamide) with the triplet regimens Rdc (lenalidomide, dexamethasone, cyclophosphamide) or Tdc (thalidomide, dexamethasone, cyclophosphamide) [12], reporting a statistically superior PFS with the quadruplet regimen (HR, 0.63; 95% CI, 0.51–0.76; *p* < 0.001). Although PFS outcomes have not been reported in other studies, they provide significant insights based on response rates. The GRIFFIN trial compared the quadruplet regimen D-RVd (daratumumab, lenalidomide, bortezomib, dexamethasone) with the triplet regimen RVd (lenalidomide, bortezomib, dexamethasone). Following autologous stem cell transplantation (ASCT), the response rate was significantly higher for the quadruplet regimen than that for the triplet regimen (99.0% vs. 91.8%; *p*= 0.016) [14]. Similarly, the GMM-HD7 trial evaluated a quadruplet regimen with isatuximab plus lenalidomide, bortezomib, and dexamethasone and compared it with the triplet regimen of lenalidomide, bortezomib, and dexamethasone. The response rate for the quadruplet regimen was higher than that for the triplet regiment (90% vs 84%; *p*= 0.049) [13]. However, the Ludwig et al. study reported no significant difference in response rates between the quadruplet regimen VTDC (bortezomib, thalidomide, dexamethasone, cyclophosphamide) and the triplet regimen VTD with approximately 100% response rates post-ASCT in both groups [15].

In summary, of the five prospective randomized studies, only two provided PFS data and both studies confirmed that the four-drug regimen significantly improved PFS as compared to the three-drug regimen. Furthermore, the remaining studies demonstrated statistically significant superior response rates with the quadruplet regimens than that with triplet regimens. Thus, while the current evidence suggests that the four-drug regimens are likely to be more effective (Supplementary Figure 1A-D), long-term follow-up data are needed for definitive conclusions.

The four-drug regimen was associated with a higher incidence of lymphopenia, leukopenia, and thrombocytopenia than the three-drug regimen. However, the severity of these adverse events was not clinically significant with no statistically significant differences in other adverse effects (Supplementary Figure 1E-K) between patients undergoing quadruplet and triplet regimens. Thus, the benefits of the four-drug regimen outweigh the potential risks.

### Patient preferences

This analysis demonstrated that the four-drug regimen was more effective than the three-drug regimen as an initial induction therapy with no significant differences in adverse events. However, the current four-drug regimen is not covered by national health insurance in Korea, creating a financial burden for patients. Overall, 138 patients responded, and despite the economic challenge, the patients preferred the four-drug regimen than the three-drug regimen (53.9% vs 44.7%).

### Recommendation

For newly diagnosed patients with transplant-eligible MM, a four-drug regimen is recommended as the initial therapy over a three-drug regimen based on its superior response rates. (Level of evidence: moderate; grade: should considered).

## Key question 2. Is continuous therapy superior to fixed-duration first-line therapy in newly diagnosed patients with transplant-ineligible MM?

A systematic literature search identified two phase 3 randomized controlled trials directly addressing this clinical question. The FIRST study was an open-label, three-arm phase 3 randomized controlled trial conducted across 246 treatment centers in 18 countries, across Europe, North America, and the Asia–Pacific region [16]. Patients were randomly assigned in a 1:1:1 ratio using a validated interactive voice response system to receive continuous lenalidomide-dexamethasone (continuous Ld), lenalidomide-dexamethasone for 18 cycles (72 weeks of treatment) (Ld18), or melphalan-prednisone-thalidomide for 12 cycles (72 weeks of treatment) (MPT). The analysis was restricted to the continuous Ld (535 patients) and Ld18 (541 patients) groups. Similarly, the MM-015 study was a phase 3, multicenter, randomized, double-blind, placebo-controlled trial conducted across 82 centers in Europe, Australia, and Israel [17]. In this trial, patients were randomly assigned in a 1:1:1 ratio to receive melphalan-prednisone-lenalidomide followed by lenalidomide maintenance therapy (MPR-R), melphalan-prednisone-lenalidomide (MPR), or melphalan-prednisone (MP). The analysis focused on MPR-R (*n* = 152) and MPR (*n* = 153) groups.

A meta-analysis of the FIRST and MM-015 trials revealed that patients continuing induction therapy had a statistically significant improvement in PFS than those administered induction therapy for a fixed duration (HR, 0.67; 95% CI, 0.58–0.77). However, no significant difference was observed in overall survival (OS) between the groups (Supplementary Figure 2A). Response rates including complete response (CR), very good partial response (VGPR) + CR, and overall response rate (ORR; CR + VGPR + PR) were analyzed. No statistically significant differences were observed in response rates between the continuous- and fixed-duration groups (Supplementary Figure 2B). The incidence of major adverse events (grades 3–4), including neutropenia, anemia, thrombocytopenia, febrile neutropenia, infections, and deep vein thrombosis, was also investigated. No significant differences were observed in the rates of these adverse events between the continuous- and fixed-duration therapy groups (Supplementary Figure 2E).

Time-based analyses of survival outcomes were conducted at 12, 18, 24, and 36 months. A statistically significant difference in PFS was observed between the fixed-duration induction and continuous therapy groups over time. While no significant difference was observed at 12 and 18 months, at 24 and 30 months the risk of disease progression was 1.41 times and 1.8 times higher, respectively, in the fixed-duration group than that in the continuous therapy group (Supplementary Figures 2C, D). No significant difference was observed in OS between the two groups at any time point.

Therefore, for newly diagnosed patients with transplant-ineligible patients MM, continuous induction therapy until disease progression, as opposed to cessation after a fixed period, results in significantly prolonged PFS without an increase in adverse effects. However, this approach did not yield an extension in OS as compared to that with fixed-duration therapy.

### Patient preferences

A survey of newly diagnosed patients with transplant-ineligible MM revealed that only 54 patients responded. Among the respondents, 59.3% indicated that they would follow their physicians’ recommendations regarding therapy continuation. Specifically, 25.9% agreed to continue induction chemotherapy, while 14.8% opposed treatment continuation.

### Recommendation

First-line therapy should be maintained for newly diagnosed patients with transplant-ineligible MM. (Level of evidence: moderate; recommendation grade: should be considered).

## Key question 3. Is initial or delayed ASCT more effective in patients with transplant-eligible MM?

A systematic search identified two relevant retrospective cohort comparative analyses [18, 19]. Both studies evaluated 5-year OS and PFS rates. However, the variation in the index dates used for survival analysis between studies was a significant limitation that introduced complexity into the comparative assessment. In the Karam et al. study [18], the index date was the date of ASCT; while, in the Leng et al. study [19], the index date was the date of the initial treatment at diagnosis. Meta-analysis of these studies revealed a trend toward poorer 5-year OS (HR, 0.72; 95% CI, 0.34–1.53) and PFS (HR, 0.82; 95% CI, 0.45–1.49) in patients who underwent delayed ASCT as compared to those who underwent ASCT within 12 months of treatment initiation. However, these differences were not significant (Supplementary Figure 3).

### Patient preferences

A survey was conducted to assess patient preferences based on the treatment outcomes. Patients were asked to prioritize survival duration or quality of life with the treatment. Most respondents indicated a preference for quality of life over survival indicators (patients prioritizing survival, 27.7%; prioritizing quality of life, 67.8%; non-respondents, 4.3%). However, the two retrospective studies reviewed did not include assessments of quality of life. Consequently, the current evidence does not facilitate recommendations incorporating patient values and preferences regarding quality of life.

### Recommendation

Based on the available evidence, immediate ASCT should be considered rather than delayed transplantation in patients with transplant-eligible MM (level of evidence: very low; recommendation grade: should be considered).

## Key question 4. Is tandem transplantation better in improving OS as to a single ASCT?

A comprehensive literature search identified five phase 3 randomized controlled trials relevant to this question. However, three of the five studies incorporated outdated induction and maintenance therapies including conventional chemotherapies (e.g., VAD therapy) and maintenance therapy (e.g., INF-a) [20–22]. The two more recent studies examined the effects of single versus tandem ASCT with various treatment regimens without directly comparing the two groups in a traditional 1:1 ratio [23, 24]. All five studies provided OS data. The earliest of these studies, conducted by Attal et al., demonstrated a statistically significant improvement in OS with tandem ASCT than that with single ASCT [20]. However, studies by Cavo et al. [21] and Mai et al. [22] reported better OS trends with single ASCT; however, statistically significant differences in OS were not observed in both studies. Overall, the meta-analysis revealed that tandem ASCT did not result in statistically significant OS improvement as compared to that with single ASCT (RR, 1.06; 95% CI, 0.91–1.22). Additional survival metrics were included in further analyses. Three studies reported relapse-free survival (RFS) [20, 23, 24], two reported event-free survival (EFS) [20, 21], and one reported PFS [24]. Meta-analysis indicated that tandem ASCT significantly improved RFS (RR, 1.21; 95% CI, 1.06–1.38; *p* = 0.005), EFS (RR, 1.46; 95% CI, 1.18–1.80; *p* = 0.0005), and PFS (HR, 0.74; 95% CI, 0.55–1.00; *p* = 0.05) as compared to that with single ASCT. Moreover, four studies evaluated VGPR or better [20–23] and the meta-analysis revealed a statistically significant superior response rate with tandem ASCT in achieving VGPR or better as compared to that with single ASCT (RR, 1.20; 95% CI, 1.06–1.35; *p* = 0.004) (Supplementary Figure 4A).

In the high-risk group, including those with high-risk cytogenetics, two studies [23, 24] reported no statistically significant difference in PFS between patients with high-risk and the overall cohort; however, a trend towards better outcomes was observed with tandem ASCT (RR, 1.39; 95% CI, 0.94–2.04). Notably, patients with 17p deletion had more pronounced benefits from tandem ASCT, with significant improvements in 5-year PFS (HR, 0.24; 95% CI, 0.09–0.67) and a trend toward improved 5-year OS (HR, 0.30; 95% CI, 0.09–1.06) (Supplementary Figure 4B).

Treatment-related mortality (TRM) was reported in two studies [20, 21]. Although the differences were not statistically significant, both studies reported a trend toward higher mortality rates in the tandem ASCT group (RR, 1.40; 95% CI, 0.69–2.81; *p* = 0.35). Moreover, more frequent infections and grade 3 or higher mucositis were observed in the tandem ASCT group (RR, 1.15; 95% CI, 0.77–1.73; *p* = 0.49; RR, 1.13; 95% CI, 0.79–1.64; *p* = 0.50). Two studies [22, 23] reported no significant differences in non-hematologic toxicities of grade ≥ 3 between the groups (RR, 1.01; 95% CI, 0.87–1.16; *p* = 0.91). One study [23] evaluated secondary cancer rates, reporting a non-significant trend toward higher rates in the tandem ASCT group (RR, 1.33; 95% CI, 0.62–2.87; *p* = 0.47) (Supplementary Figure 4C).

In summary, the meta-analysis of these five phase 3 randomized trials did not reveal a statistically significant difference in OS between the tandem and single ASCT groups. However, the analysis revealed trends toward higher TRM and secondary cancers in the tandem ASCT group. Consequently, tandem ASCT is generally not recommended for patients with MM. Nevertheless, for patients with high-risk, especially those with 17p deletions, tandem ASCT may offer substantial improvements in the PFS and OS. As patients with high-risk typically have poorer outcomes, tandem ASCT may be considered in some cases based on the clinician discretion.

### Patient preferences

A survey was conducted with the following question: “For the treatment of MM, undergoing tandem ASCT may potentially extend survival compared to undergoing the procedure once, but it could also increase the risk of complications such as mucositis, infections, and mortality. In this case, would you choose to undergo tandem ASCT?” The survey was administered to 141 patients from 17 domestic institutions. Among the respondents, 14.9% preferred tandem ASCT, 13.5% opposed it, and 35.5% preferred to defer their decision to their physician’s judgment. Overall, 51 non-respondents (36.1%) were observed. As only 21 patients (14.9%) actively opted for tandem ASCT, most patients with MM likely do not favor tandem ASCT.

### Recommendation

Current data indicates that tandem ASCT does not provide a significant survival advantage over single ASCT, and is associated with increased toxicity. Therefore, tandem ASCT is not recommended for patients with MM. Nonetheless, this should be considered on a limited basis in patients with high-risk MM. (Level of evidence: low; recommendation grade: may be considered).

## Key question 5. Does maintenance therapy after ASCT improve survival in patients with MM?

A comprehensive analysis of six prospective randomized phase III trials was conducted to evaluate the impact of maintenance therapy post-ASCT in patients with MM. Among these, four studies assessed lenalidomide for maintenance therapy (CALGB 100104, IFM 2005, GIMEMA RVMM-PI-209, Myeloma XI) [25–29]; while, the remaining two evaluated ixazomib (TOURMALINE-MM3) [30] and daratumumab (CASSIOPEIA) [31] as maintenance drugs.

In the CALGB 100104 trial, lenalidomide maintenance therapy significantly improved both PFS (HR, 0.57; 95% CI, 0.46–0.71) and OS (HR, 0.61; 95% CI, 0.46–0.81) as compared to that with placebo [26, 28]. Similarly, the IFM 2005 study reported a significant enhancement in PFS with lenalidomide maintenance therapy (HR, 0.50; 95% CI, 0.35–0.71) as compared to that with placebo; however, no significant difference was observed in OS between the two groups (HR, 1.25; 95% CI, 0.83–1.88) [25]. The GIMEMA RVMM-PI-209 trial reported a significant PFS prolongation with lenalidomide maintenance therapy (HR, 0.42; 95% CI, 0.24–0.73) as compared to that with placebo; however, no significant difference was observed in OS between the groups (HR, 0.62; 95% CI, 0.24–1.60) [27]. In the Myeloma XI study, lenalidomide maintenance significantly improved both PFS (HR, 0.48; 95% CI, 0.40–0.58) and OS (HR, 0.69; 95% CI, 0.52–0.92) as compared to that with placebo [29]. The TOURMALINE-MM3 study reported significantly extended PFS with ixazomib maintenance (HR, 0.72; 95% CI, 0.58–0.89) as compared to that with placebo [30]. Furthermore, the CASSIOPEIA trial reported that daratumumab maintenance therapy significantly improved PFS (HR, 0.53; 95% CI, 0.42–0.67) as compared to that with placebo [31]. A meta-analysis of the CALGB 100104, IFM 2005, GIMEMA RVMM-PI-209, Myeloma XI, TOURMALINE-MM3, and CASSIOPEIA studies revealed that maintenance therapy was associated with a significant improvement in PFS as compared to that with placebo (HR, 0.55; 95% CI, 0.50–0.61) (Supplementary Figure 5A). Moreover, a meta-analysis of the CALGB 100104, IFM 2005, GIMEMA RVMM-PI-209, and Myeloma XI studies revealed that maintenance therapy significantly enhanced OS as compared to that with placebo (HR, 0.73; 95% CI, 0.61–0.87) (Supplementary Figure 5B).

Maintenance therapy was significantly associated with increased incidences of grade 3–4 neutropenia, grade 3–4 thrombocytopenia, infections, and secondary malignancies as compared to that with no maintenance therapy. However, no significant difference was observed in the incidence of febrile neutropenia between the groups (Supplementary Figure 5C).

The meta-analysis confirmed that maintenance therapy significantly improved PFS. Among the four studies reporting OS, two revealed a statistically significant increase in OS with maintenance therapy as compared to that with placebo while, the other two studies did not. Nonetheless, a pooled meta-analysis of these studies confirmed a significant improvement in OS with maintenance therapy. Despite the association between maintenance therapy and increased risks of neutropenia, thrombocytopenia, infections, and secondary malignancies, the substantial increase in both PFS and OS suggest that the therapeutic benefits of maintenance therapy outweigh the associated risks.

### Patient preferences

A survey was conducted among patients with newly diagnosed MM, and 90 patients responded that 52.8% of the participants preferred adhering to their physician’s recommendations regarding maintenance therapy, 46.0% agreed to maintenance therapy, and 2.2% did not want to undergo the therapy.

### Recommendation

Maintenance therapy after ASCT significantly enhanced PFS and OS in patients with MM. Therefore, maintenance therapy was recommended. (Level of evidence: high; recommendation grade: should be considered).

## Key question 6. Does treatment during biochemical relapse improve survival as compared to treatment during symptomatic relapse in relapsed MM?

A review of the literature identified five retrospective studies addressing this question [32–36]. OS was evaluated in all five studies with significant improvement in OS for patients administered salvage therapy during biochemical relapse (HR, 0.42; 95% CI, 0.21–0.64). PFS was assessed in two studies [32, 34], and one study [33] used the time to next treatment (TTNT) as a metric. Across all studies, treatment initiated at biochemical relapse was associated with improved PFS (HR, 0.55; 95% CI, 0.54–0.70) (Supplementary Figure 6). One study included subgroup analysis to evaluate patients with and without extramedullary disease at relapse [34] and revealed that regardless of the presence of extramedullary disease, patients with clinical symptoms had lower survival rates than those treated at biochemical relapse. However, some studies stratified patients according to relapse type (biochemical vs. clinical) during analysis, necessitating careful interpretation of the results. Furthermore, lead-time bias associated with initiating treatment at biochemical relapse may exist; however, no studies have explicitly assessed this effect, warranting caution when interpreting survival benefits.

No additional adverse effects have been reported with treatment initiation at biochemical relapse as compared to that at clinical relapse. Owing to the potential adverse effects of the current therapeutic agents, the survival benefits observed with earlier intervention at biochemical relapse likely outweigh the associated risks.

### Patient preferences

Analysis of patient preferences revealed that 140 of the 141 patients (99.3%) favored initiating treatment at biochemical relapse as comparted to waiting until clinical relapse, highlighting a strong inclination toward early intervention.

### Recommendation

For patients with relapsed MM, treatment initiation at biochemical relapse is recommended, rather than at the clinical symptom onset. (Level of evidence: low; recommended grade: should be considered).

## Key question 7. Is retreatment with previously effective agents a viable approach for relapsed MM?

A literature review identified 10 retrospective studies relevant to this question [37–46]. These studies included studies on lenalidomide (*n* = 1), bortezomib (*n* = 8), and carfilzomib (*n* = 1) reuse. In these studies, the original dosage was used for lenalidomide and bortezomib; while, an increased dosage as compared to that at the initial treatment was used for carfilzomib. In the absence of studies directly comparing treatment outcomes of the intervention and control groups specific to this clinical question, a representative control group was selected from phase 3 randomized controlled trials on recommended drugs for relapsed patients to assess the efficacy of the intervention [47–67]. The ORR for patients retreated with previously used agents was 57% (95% CI, 42–71%). The CR, VGPR, and PR rates were 9% (95% CI, 4–15%), 6% (95% CI, 2–11%), and 37% (95% CI, 26–47%), respectively (Supplementary Figure 7A). While, for patients administered new therapies, the ORR, CR, VGPR, and PR rates were 65% (95% CI, 60–70%), 14% (95% CI, 11–17%), 26% (95% CI, 21–30%), and 30% (95% CI, 23–37%), respectively (Supplementary Figure 7B). Although the ORR for retreatment was considered acceptable (57% vs. 65%), the CR (9% vs. 14%) and VGPR (6% vs. 26%) rates were lower. PFS was analyzed in four of the ten studies, with a median PFS of 6.32 months (95% CI, 4.57–8.07 months), and OS was assessed in two studies with a median OS of 14.35 months (95% CI, 8.82–19.88 months) (Supplementary Figure 7C).

Among the 10 studies, 8 focused specifically on bortezomib reuse, prompting further analysis of this subgroup [F-I]. In patients retreated with bortezomib, the ORR was 63% (95% CI, 51–76%), with CR, VGPR, and PR rates of 13% (95% CI, 5–22%), 7% (95% CI, 1–12%), and 41% (95% CI, 36–46%), respectively. While, for patients administered bortezomib for the first time, the ORR, CR, VGPR, and PR rates were 57% (95% CI, 49–66%), 7% (95% CI, 5–9%), 20% (95% CI, 17–23%), and 33% (95% CI, 30–35%), respectively (Supplementary Figure 7D). While direct comparison is challenging, bortezomib reuse was associated with a higher ORR and CR rate (63% vs. 57%; 13% vs. 7%) but a lower VGPR rate (7% vs. 20%).

Regarding adverse events, the incidence of leukopenia, thrombocytopenia, and anemia in patients retreated with previously effective agents were 5% (95% CI: 0–11%), 15% (95% CI: 3–27%), and 4% (95% CI: 1–8%), respectively. While, the incidence of leukopenia, thrombocytopenia, and anemia rates in patients administered new therapies were 25% (95% CI, 19–30%), 22% (95% CI, 18–25%), and 17% (95% CI, 15–18%), respectively. These findings suggest that the hematological side effects of retreatment were within acceptable limits. For non-hematological side effects, the incidence rates of infections, neuropathy, and pneumonia in patients retreated with previously effective agents were 1% (95% CI, 0–2%), 4% (95% CI, 0–8%), and 5% (95% CI, 2–8%), respectively. However, new therapies were associated with higher rates of infection (12%, 95% CI, 7–16%), neuropathy (4%, 95% CI, 2–5%), and pneumonia (10%, 95% CI, 9–12%) (Supplementary Figure 7E). These data indicate that the non-hematological side effects of retreatment were also within acceptable ranges.

### Patient preferences

A survey was administered to 141 patients with MM to assess their preferences for the reuse of previously administered treatments upon disease relapse. The survey specifically examined patient expectations and the factors deemed important in this context. Regarding the minimum acceptable treatment outcome when reusing prior therapy, 133 patients responded, with 115 patients (81.5%) indicating a preference for an ORR of 50% or higher. Overall, 140 patients (99.3%) provided information on the factors considered important when reusing previously administered treatments. The factors identified included cost of therapy (8.5%), side effects (18.4%), response rate (27.0%), survival rate (19.1%), and the physician’s judgment (32.6%).

### Recommendation

In cases with no other effective treatment available for patients with relapse, the reuse of a previously effective drug may represent a viable therapeutic option. (Level of evidence: very low; recommendation grade: should be considered).

## Key question 8. Is the use of antibiotics or antivirals effective for preventing infections during initial induction therapy in patients with newly diagnosed MM?

A comprehensive literature search to evaluate the role of prophylactic antibiotics in patients newly diagnosed with MM revealed five relevant studies. Of these, one was a randomized phase 3 trial [68], two were retrospective cohort comparison studies [69, 70], and two were prospective phase 2 trials [71, 72]. These studies used different treatment regimens, follow-up durations, and participant characteristics, making uniform analysis difficult. Nonetheless, a meta-analysis revealed that prophylactic antibiotic use significantly reduced infection rates in both prospective and retrospective studies. The relative risk (RR) for infection reduction in the prospective studies was 0.47 (95% CI, 0.22–0.99) in the randomized controlled trial, and 0.56 (95% CI, 0.39–0.81) in the non-randomized studies (Supplementary Figure 8-1A).

Regarding the adverse effects associated with prophylactic antibiotic use, a meta-analysis of prospective studies [68, 71, 72] revealed no significant difference in the overall occurrence of adverse events, including those of grade 3 or higher. Among the retrospective studies, only one study [69] reported adverse events with four cases in the treatment group and none in the control group. However, owing to the low incidence of adverse events and the absence of reports in the control group, the data on adverse events were likely insufficient owing to the limitations of the retrospective study design (Supplementary Figure 8-1B).

Five studies were also evaluated to assess the effectiveness of prophylactic antiviral agents in reducing the incidence or reactivation of herpes zoster [73–77]. A meta-analysis of these studies revealed that the use of prophylactic antiviral agents significantly reduced herpes zoster incidence (RR, 0.04; 95% CI, 0.01–0.29) (Supplementary Figure 8–2).

### Patient preferences

All patients provided their responses. Of the respondents, 98.6% expressed interest in following up or relying on their physician’s decision for prophylactic antibiotic use. Owing to the significant impact of infections on the morbidity and mortality of patients with MM, and their willingness to adhere to their physicians’ recommendations, clinicians must carefully select prophylactic antibiotics and antiviral agents for appropriate patient groups considering the efficacy and potential adverse effects of these prophylactic measures.

The data revealed no significant differences in the adverse effects between the groups administered prophylactic antibiotics and those that did not. However, prophylactic antibiotic use was associated with a significant reduction in the incidence of infections. Similarly, prophylactic antiviral agents substantially lowered the risk of herpes zoster infection. Therefore, based on the minimal adverse effect profile of prophylactic antibiotics and their effectiveness in reducing infection rates, both prophylactic antibiotics and antiviral agents must be used to prevent infections during the initial induction therapy in patients with newly diagnosed MM.

### Recommendation

Prophylactic antibiotics are recommended during initial therapy for newly diagnosed MM (level of evidence: moderate; recommendation grade: should be considered).

Prophylactic antiviral agents are recommended during the initial therapy for newly diagnosed MM (level of evidence: very low, recommendation grade: should be considered).

## Key question 9. Is the use of bone resorption inhibitors effective in MM treatment?

Overall, 11 prospective randomized controlled trials assessing the effect of pamidronate and zoledronate were identified [78–88]. No direct randomized controlled trials comparing denosumab with a placebo or non-treatment group were available. Therefore, an indirect comparison was conducted via a large-scale, phase 3 randomized controlled trials, assessing the effect of denosumab against zoledronate in a 1:1 ratio [89]. A meta-analysis of these 11 prospective studies confirmed that both pamidronate and zoledronate significantly reduced the incidence of skeletal-related events (SREs) as compared to the placebo or no treatment in patients with MM (Supplementary Figure 9A). Furthermore, both agents resulted in statistically significant improvements in OS and PFS rates. In a study involving 1,718 patients with MM across 29 countries, denosumab was observed to be non-inferior to zoledronate in reducing the SRE incidence of SREs based on OS and PFS outcomes (Supplementary Figure 9B, C).

Although pamidronate and zoledronate were associated with increased gastrointestinal side effects and jaw osteonecrosis (ONJ), these differences were not statistically significant (Supplementary Figure 9D, E). Furthermore, no significant difference was observed between denosumab and zoledronate use in terms of grade 3 or higher adverse events, including ONJ. However, denosumab treatment was associated with a significantly higher hypocalcemia incidence (Supplementary Figure 9F).

### Patient preferences

A preference survey conducted among patients with MM revealed that 97 of the 135 respondents (71.8%) prioritized quality of life over survival prolongation. As pamidronate, zoledronate, and denosumab are effective in delaying or preventing SREs, these therapies are likely to enhance the quality of life of patients with MM and be preferred by patients.

### Recommendation

The use of pamidronate and zoledronate is recommended to prevent and delay SREs in patients with active MM. (Level of evidence: moderate; recommendation grade: recommended).

Denosumab may be considered for the prevention and delay of SREs in patients with active MM owing to its demonstrated non-inferiority to zoledronate. (Level of evidence: low; recommendation grade: should be considered).

## Key question 10. Are fluorodeoxyglucose positron emission tomography/computed tomography (FDG PET/CT) or magnetic resonance imaging (MRI) useful for predicting prognosis of patients with newly diagnosed MM?

The studies included in the meta-analysis are summarized in Supplementary Table 10-1A [90–101] and 10-1B [100, 102–110]. The meta-analysis revealed that the sensitivity and specificity of PET/CT for predicting PFS were 56% and 64%, respectively. MRI had a higher sensitivity (83%), although its specificity was lower (39%). Regarding the OS, PET/CT had a sensitivity and specificity of 62% and 63%, respectively; while, MRI had a sensitivity and specificity of 78% and 42%, respectively. Across all studies, the number of focal lesion (FL) detected by PET/CT (FL > 3 vs. FL ≤ 3) was statistically significant in predicting both PFS and OS. Similarly, diffuse MRI patterns and multiple focal lesions were significantly associated with prognosis. Although the meta-analysis did not reveal whether PET/CT or MRI was superior for prognostic prediction, PET/CT studies consistently used a threshold of three or four focal lesions to define the number of lesions. While, MRI studies had variability in reporting diffuse involvement and the number of focal lesions (Supplementary Figure 10A, B). Consequently, PET/CT is recommended for patients newly diagnosed with MM. However, owing to the higher sensitivity of MRI, it may be advantageous in assessing bone involvement in MM. Furthermore, bone involvement may serve as a treatment initiation criterion in patients with asymptomatic MM, supporting the use of MRI when clinically indicated.

A single PET/CT scan exposes the patient to radiation at < 10 mSv, generally considered safe. MRI, while causing discomfort owing to the length of the scan, does not result in radiation exposure. Although both modalities are expensive, they are manageable under national health insurance coverage.

### Patient preferences

A patient survey on the adverse effects, costs, and clinical implications of PET/CT and MRI, including access to valuable prognostic information without significant effect of these imaging studies on treatment plans, revealed that 139 of 141 respondents (98.6%) desired PET/CT or MRI. Among these, 40 patients (28.4%) preferred PET/CT and 33 patients (23.4%) preferred MRI. Importantly, 59 patients (41.8%) expressed a desire to undergo both PET/CT and MRI. These results indicate that despite the potential adverse effects, nearly all patients had a strong preference for imaging.

### Recommendation

In newly diagnosed MM, both FDG PET/CT and MRI have significant prognostic value. Therefore, both imaging modalities are recommended to predict the prognosis of these patients. (Level of evidence: very low; recommendation grade: should be considered).

The higher specificity of PET/CT is particularly valuable for diagnosing extramedullary plasmacytoma; while, the higher sensitivity of the MRI is valuable for detecting bone involvement in MM. This is particularly critical in asymptomatic MM with bone involvement being as a key criterion for initiating treatment.

## Key question 11. Is the assessment of MRD useful in patients with newly diagnosed MM?

The 2016 International Myeloma Working Group (IMWG) 2016 guidelines established the criteria for evaluating MRD in patients with MM. This meta-analysis included studies published after 2016 using MRD testing with a sensitivity of at least 10^-4^ (0.01%). Various MRD assessment methods have been used including multicolor flow cytometry, next-generation flow cytometry, and next-generation sequencing. The timing of the MRD assessments and treatment regimens varied across studies, encompassing both ASCT and chemotherapy. Overall, 29 studies, representing 32 research protocols, were included in the PFS analysis [111–136]. The results revealed a significant extension of PFS in patients with a MRD-negative status(HR, 0.28; 95% CI, 0.23–0.33) (Supplementary Figure 11A). This extension was consistent irrespective of MRD sensitivity being defined as < 10^-5^ or 10^–4^–10^–5^ (HR, 0.27; 95% CI, 0.20–0.34; HR, 0.31; 95% CI, 0.23–0.38, respectively) (Supplementary Figure 11B). Both MRD-negative status after chemotherapy (HR, 0.21; 95% CI, 0.17–0.26) and post-ASCT (HR, 0.33; 95% CI, 0.19–0.48) were associated with significantly extended PFS (Supplementary Figure 11C). Furthermore, assessing MRD measurement timing post-treatment revealed that MRD-negative status within 6 months (HR, 0.44; 95% CI, 0.34–0.54), between 6 to 12 months (HR, 0.18; 95% CI, 0.11–0.26), and beyond 12 months (HR, 0.14; 95% CI, 0.10–0.18) was significantly correlated with extended PFS (Supplementary Figure 11D).

The meta-analysis of MRD assessment for OS prediction included 15 research protocols from 14 studies [111, 112, 114, 115, 119, 122–124, 128, 130, 131, 137–139]. The results revealed a significant extension in OS among patients with a MRD-negative status (HR, 0.36; 95% CI, 0.30–0.43) (Supplementary Figure 11E). This OS extension was consistent in studies with MRD sensitivity thresholds <–10^5^ (HR, 0.34; 95% CI, 0.22–0.46) and 10^–4^–10^–5^ (HR, 0.38; 95% CI, 0.29–0.46) (Supplementary Figure 11F). Moreover, MRD-negative status detected after ASCT or chemotherapy was significantly associated with an extended OS (HR, 0.37; 95% CI, 0.29–0.45) (Supplementary Figure 11G). Consistent OS benefits were observed across all post-treatment MRD measurement time points (under 6 months, 6 to 12 months, and over 12 months), with MRD negativity correlating with significantly improved OS (HR, 0.48; 95% CI, 0.28–0.67) (Supplementary Figure 11H).

### Patient preferences

Of the 138 respondents, 47.1% agreed to undergo MRD testing; while, 6.5% disagreed. Moreover, 46.4% of the participants indicated that would adhere to their physicians’ recommendations. As responses favoring physician discretion could be interpreted as positive, 93.5% of patients were willing to participate in MRD testing.

### Recommendation

MRD assessment is recommended for patients with newly diagnosed MM. (Level of evidence: very low; recommendation grade: should be considered).

MRD assessment should be performed using multicolor flow cytometry, next-generation flow cytometry, or next-generation sequencing with a sensitivity of at least 10^-4^ during clinical evaluation following treatment.

## Key question 12. Is immediate treatment initiation beneficial in patients with high-risk smoldering MM?

A systematic review of the literature identified three high-quality randomized phase III trials [140–142] investigating early treatment initiation in high-risk patients with asymptomatic MM. None of these studies used the most recent IMWG diagnostic criteria [143] for patient selection. Specifically, all studies included patients with characteristics including ≥ 60% clonal bone marrow plasma cell infiltration, an involved to uninvolved free light-chain ratio ≥ 100, or more than one MRI-detectable bone lesion ≥ 5 mm. These characteristics are now used to diagnose active MM. However, these updated criteria affect only a subset of asymptomatic MM cases [144]. The Lonial et al. study [142] confirmed MM diagnosis via MRI; however, the definitions of high-risk groups varied between studies.

All three trials reported PFS defined as the time to progression to active MM. The meta-analysis revealed a significant increase in PFS in the treatment group (HR, 0.31; 95% CI, 0.23 0.41). Moreover, two trials [140, 142] evaluated lenalidomide-based interventions, currently widely used in MM treatment, both reporting substantial PFS extension (Mateos et al.: HR, 0.28; 95% CI, 0.18–0.44; Lonial et al.: HR, 0.28; 95% CI, 0.12–0.62).

Only Mateos et al. [145] have reported this outcome in terms of OS. After a median follow-up of 12 years, the intervention group had a significant improvement in OS (median not reached vs. 8.5 years; HR, 0.57; 95% CI, 0.34–0.95). However, the inability to exclude active MM at baseline owing to the lack of MRI and other imaging modalities, as well as the use of multiparametric flow cytometry (a less common method) for identifying patients with high-risk could be concerning. Statistically, the upper limit of the 95% CI for HR approached 1 with no significant difference in survival observed between the groups following progression to active MM (*p* = 0.96). As OS data were limited to a single study, these recommendations remain conditional (Supplementary Figure 12A).

The analysis of adverse events associated with the treatment of asymptomatic MM was challenging owing to inconsistent definitions and reporting across trials. A trend towards increased grade 3–5 AEs was observed in the treatment group; however, this increase was not statistically significant. The Lonial et al. trial [142] with lenalidomide reported significant increase in adverse events with a RR of 9.45 (95% CI, 3.56–25.07) (Supplementary Figure 12B). Overall, the trials provided limited details on adverse events, potentially reflecting the well-established safety profiles of drugs, widely administered to patients with MM. In the Mateos et al. [145] study, one treatment-related death (1.7% due to respiratory infection) was reported among 57 patients in the treatment group, while in the Lonial et al. [142] study, one treatment-related death (0.7% due to pulmonary embolism) was reported among 134 patients in the treatment group.

### Patient preferences

Of the 137 respondents, 27.7% preferred observation without treatment following the diagnosis of asymptomatic MM; while, 72.3% favored treatment initiation. However, the survey focused on the progression to MM as the primary endpoint and did not include data on survival outcomes. Therefore, patients and healthcare providers need to engage in comprehensive discussions regarding the risks and benefits of early treatment initiation.

### Recommendation

For patients with high-risk asymptomatic MM, immediate treatment initiation is recommended to extend PFS and OS. (Evidence Level: low; recommendation grade: should considered).

## Supplementary Information

Supplementary Material 1.

## Data Availability

No datasets were generated or analysed during the current study.
