# Correction: Evidence‑based Korean guidelines for the clinical management of multiple myeloma: addressing 12 key clinical questions

**DOI:** 10.1007/s44313-025-00074-6

**Published:** 2025-04-08

**Authors:** Sung‑Hoon Jung, Youngil Koh, Min Kyoung Kim, Jin Seok Kim, Joon Ho Moon, Chang‑Ki Min, Dok Hyun Yoon, Sung‑Soo Yoon, Je‑Jung Lee, Chae Moon Hong, Ka‑Won Kang, Jihyun Kwon, Kyoung Ha Kim, Dae Sik Kim, Sung Yong Kim, Sung‑Hyun Kim, Yu Ri Kim, Young Rok Do, Yeung‑Chul Mun, Sung‑Soo Park, Young Hoon Park, Ho Jin Shin, Hyeon‑Seok Eom, Sang Eun Yoon, Sang Mee Hwang, Won Sik Lee, Myung‑won Lee, Jun Ho Yi, Ji Yun Lee, Ji Hyun Lee, Ho Sup Lee, Sung‑Nam Lim, Jihyang Lim, Ho‑Young Yhim, Yoon Hwan Chang, Jae‑Cheol Jo, Jinhyun Cho, Hyungwoo Cho, Yoon Seok Choi, Hee jeong Cho, Ari Ahn, Jong Han Choi, Hyun Jung Kim, Kihyun Kim

**Affiliations:** 1 Department of Hematology‑Oncology, Chonnam National University Hwasun Hospital, Chonnam National University Medical School, Hwasun, Republic of Korea; 2 Division of Hematology‑Oncology, Department of Internal Medicine, Seoul National University Hospital, Seoul National University College of Medicine, Seoul, Republic of Korea; 3 Division of Hemato‑Oncology, Department of Internal Medicine, Yeungnam University College of Medicine, Daegu, Republic of Korea; 4 Division of Hematology, Department of Internal Medicine, Yonsei University College of Medicine, Severance Hospital, Seoul, Republic of Korea; 5 Department of Hematology/Oncology, Kyungpook National University Hospital, School of Medicine, Kyungpook National University, Daegu, Republic of Korea; 6 Seoul St. Mary’s Hospital, College of Medicine, The Catholic University of Korea, Seoul, Republic of Korea; 7 Department of Oncology, Asan Medical Center, University of Ulsan College of Medicine, Seoul, Republic of Korea; 8 Department of Nuclear Medicine, School of Medicine, Kyungpook National University, Daegu, Republic of Korea; 9 Division of Hematology‑Oncology, Department of Internal Medicine, Korea University College of Medicine, Seoul, Republic of Korea; 10 Department of Internal Medicine, Hematology and Oncology, College of Medicine, Chungbuk National University, Cheongju, Republic of Korea; 11 Division of Hematology and Oncology, Department of Internal Medicine, Soonchunhyang University Seoul Hospital, Seoul, Republic of Korea; 12 Division of Oncology & Hematology, Department of Internal Medicine, Korea University Guro Hospital, Seoul, Republic of Korea; 13 Hematology & Oncology, Konkuk University Medical Center, Konkuk University School of Medicine, Seoul, Republic of Korea; 14 Division of Hematology‑Oncology, Department of Internal Medicine, Dong-A University College of Medicine, Busan, Republic of Korea; 15 Division of Hematology‑Oncology, Department of Internal Medicine, Dongsan Medical Center, Keimyung University School of Medicine, Daegu, Republic of Korea; 16 Department of Internal Medicine, Ewha Womans University College of Medicine, Seoul, Republic of Korea; 17 Division of Hematology‑Oncology, Department of Internal Medicine, Pusan National University Hospital, Pusan National University School of Medicine, Busan, Republic of Korea; 18 Department of Hematology‑Oncology, Center for Hematologic Malignancy, National Cancer Center, Goyang, Republic of Korea; 19 Division of Hematology‑Oncology, Department of Medicine, Samsung Medical Center, Sungkyunkwan University School of Medicine, 81 Irwon‑Ro, Gangnam‑Gu, Seoul, Republic of Korea; 20 Department of Laboratory Medicine, Seoul National University Bundang Hospital, Seoul National University College of Medicine, Seongnam, Republic of Korea; 21 Department of Internal Medicine, Hematology, Inje University Busan Paik Hospital, Busan, Republic of Korea; 22 Division of Hematology and Oncology, Department of Internal Medicine, Chungnam National University Hospital, Daejeon, Republic of Korea; 23 Division of Hematology‑Oncology, Department of Medicine, Chung-Ang University, Seoul, Republic of Korea; 24 Division of Hematology and Medical Oncology, Department of Internal Medicine, Seoul National University Bundang Hospital, Seoul National University College of Medicine, Seongnam, Republic of Korea; 25 Department of Internal Medicine, Kosin University College of Medicine, Kosin University Gospel Hospital, Busan, Republic of Korea; 26 Department of Internal Medicine, Inje University College of Medicine, Haeundae Paik Hospital, Busan, Republic of Korea; 27 Department of Laboratory Medicine, Eunpyeong St. Mary’s Hospital, College of Medicine, The Catholic University of Korea, Seoul, Republic of Korea; 28 Department of Internal Medicine, Jeonbuk National University Medical School, Jeonju, Republic of Korea; 29 Department of Laboratory Medicine, Seoul National University Hospital, Seoul National University College of Medicine, Seoul, Republic of Korea; 30 Department of Hematology and Oncology, Ulsan University Hospital, University of Ulsan College of Medicine, Ulsan, Republic of Korea; 31 Division of Hematology‑Oncology, Department of Internal Medicine, Inha University Hospital, Inha University School of Medicine, Incheon, Republic of Korea; 32 Department of Laboratory Medicine, College of Medicine, Incheon St. Mary’s Hospital,, The Catholic University of Korea, Seoul, Republic of Korea; 33 Department of Endocrine and Metabolism Medicine, Konkuk University Medical Center, Seoul, Republic of Korea; 34 Institute for Evidence‑Based Medicine, Cochrane Korea College of Medicine, Korea University, Seoul, Republic of Korea

**Correction: Blood Res 60, 9 (2025)**

**https://doi.org/10.1007/s44313-025-00055-9**

On page 11, within the recommendations section, the second paragraph incorrectly reads: “MRD assessment should be performed using multicolor flow cytometry, next-generation flow cytometry, or next-generation sequencing with a sensitivity of at least **10 units** during clinical evaluation following treatment” but it should have read “MRD assessment should be performed using multicolor flow cytometry, next-generation flow cytometry, or next-generation sequencing with a sensitivity of at least **10**^**–4**^ during clinical evaluation following treatment.”

The original article has been updated [1].
