# Supplementary material for: Evidence-based Korean guidelines for the clinical management of multiple myeloma: addressing 12 key clinical questions
Source: Blood Res. 2025 Feb 4;60(1):9. doi: 10.1007/s44313-025-00055-9 (PMC11794900; doi:10.1007/s44313-025-00055-9)

**Key Question 1. Is a four-drug regimen more effective than a three-drug regimen as induction therapy in transplant-eligible multiple myeloma**

**Table 1. Summary of Findings table**

| **Outcomes** | **Illustrative comparative risks* (95% CI)** | | **Relative effect (95% CI)** | **No of Participants (studies)** | **Quality of the evidence (GRADE)** | **Comments** |
| --- | --- | --- | --- | --- | --- | --- |
|  | Assumed  risk | Corresponding  risk |  |  |  |  |
| **PFS** | **236 per 1000** | **144 per 1000** (123 to 172) | **HR 0.58**  (0.49 to 0.70) | 1658 (2 studies) | ⊕⊕⊕⊝ **moderate** |  |
| **PR** | **899 per 1000** | **935 per 1000** (908 to 953) | **RR 1.04**  (1.01 to 1.06) | 2599 (5 studies) | ⊕⊕⊕⊝ **moderate** |  |
| **≥ VGPR** | **740 per 1000** | **836 per 1000** (799 to 866) | **RR 1.13**  (1.08 to 1.17) | 2599 (5 studies) | ⊕⊕⊕⊝ **moderate** |  |
| **MRD (10^-5^)** | **400 per 1000** | **585 per 1000** (536 to 637) | **RR 1.46**  (1.34 to 1.59) | 2318 (5 studies) | ⊕⊕⊕⊕ **high** |  |
| **ANEMIA** | **61 per 1000** | **81 per 1000** (56 to 116) | **RR 1.32**  (0.92 to 1.90) | 1725 (4 studies) | ⊕⊕⊕⊝ **moderate** |  |
| **DIARRHEA** | **22 per 1000** | **41 per 1000** (18 to 93) | **RR 1.85**  (0.82 to 4.16) | 969 (2 studies) | ⊕⊕⊕⊝ **moderate** |  |
| **INFECTION**  **(ZOSTER)** | **95 per 1000** | **123 per 1000** (85 to 179) | **RR 1.30**  (0.90 to 1.89) | 969 (2 studies) | ⊕⊕⊕⊝ **moderate** |  |
| **LYMPHOPENIA** | **155 per 1000** | **197 per 1000** (165 to 238) | **RR 1.27**  (1.06 to 1.53) | 2031 (4 studies) | ⊕⊕⊕⊝ **moderate** |  |
| **NEUROPATHY** | **68 per 1000** | **65 per 1000** (49 to 86) | **RR 0.96**  (0.72 to 1.28) | 2799 (5 studies) | ⊕⊕⊕⊝ **moderate** |  |
| **NEUTROPENIA** | **148 per 1000** | **237 per 1000** (203 to 277) | **RR 1.60**  (1.37 to 1.87) | 2799 (5 studies) | ⊕⊕⊕⊝ **moderate** |  |
| **THROMBOCYTOPENIA** | **57 per 1000** | **98 per 1000** (74 to 129) | **RR 1.71**  (1.29 to 2.26) | 2680 (5 studies) | ⊕⊕⊕⊝ **moderate** |  |
| *The basis for the **assumed risk** (e.g. the median control group risk across studies) is provided in footnotes. The **corresponding risk** (and its 95% confidence interval) is based on the assumed risk in the comparison group and the relative effect of the intervention (and its 95% CI). CI, confidence interval; RR, risk ratio; HR, hazard ratio; PFS progression free survival, PR, partial response; VGPR, very good partial response; MRD, minimal residual disease. | | | | | | |

**Figure 1. Results of meta-analysis**

**A) Progression free survival**


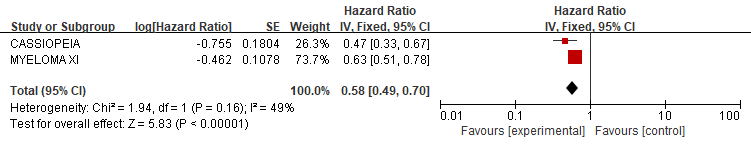


**B) Partial response**


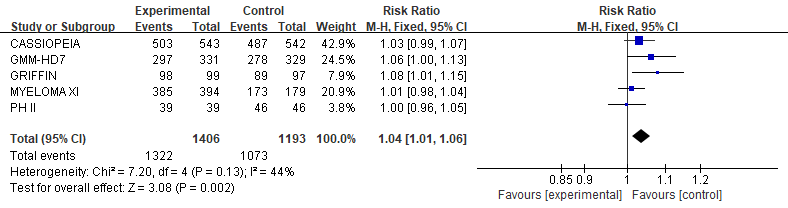


**C) Very good partial response**


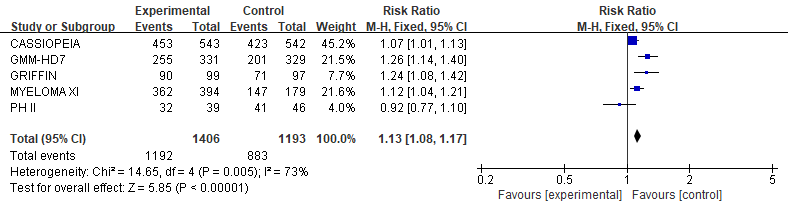


**D) Minimal residual disease**


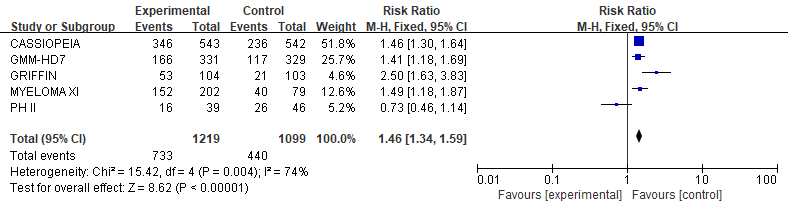


**E) Anemia**


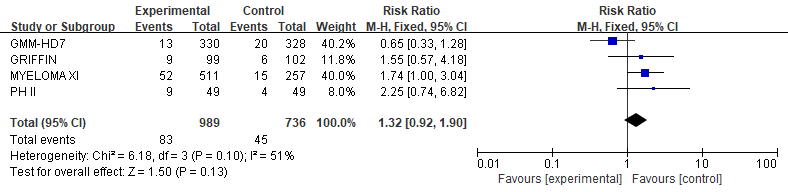


**F) Diarrhea**


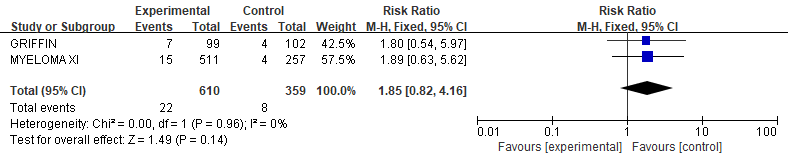


**G) Infection**


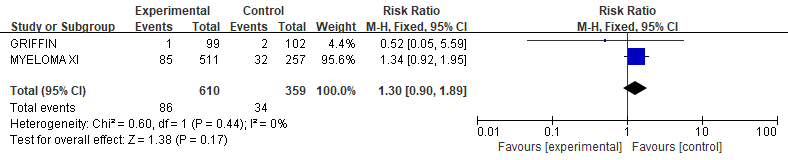


**H) Lymphopenia**


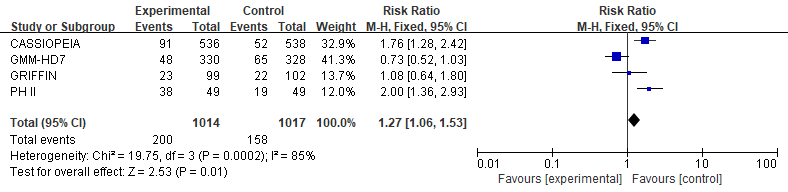


**I) Neuropathy**


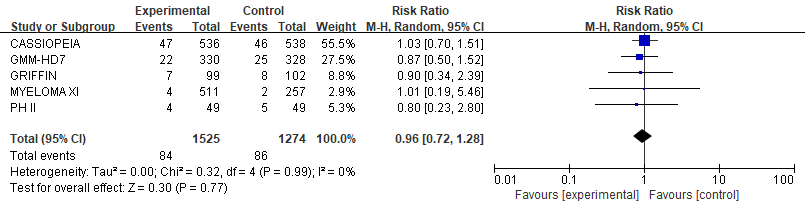


**J) Neutropenia**


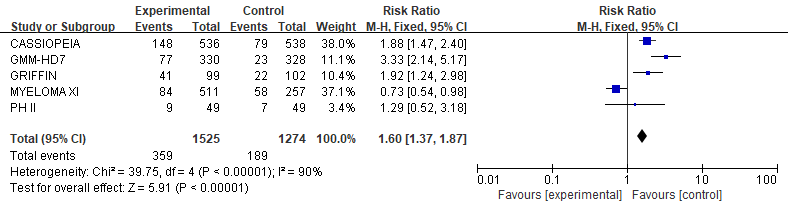


**K) Thrombocytopenia**


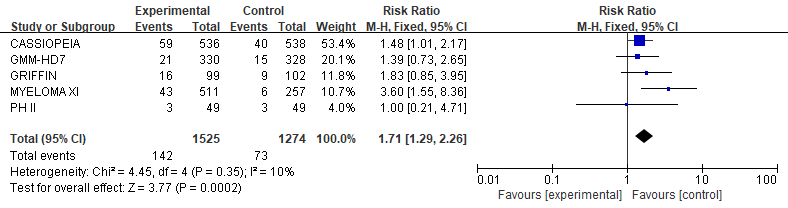


**Key Question 2. Is continuous therapy superior to a fixed duration of induction therapy in newly diagnosed transplant-ineligible multiple myeloma?**

**Table 2. Summary of Findings table**

| **Outcomes** | **Illustrative comparative risks* (95% CI)** | | **Relative effect (95% CI)** | **No of Participants (studies)** | **Quality of the evidence (GRADE)** | **Comments** |
| --- | --- | --- | --- | --- | --- | --- |
|  | **Assumed risk** | **Corresponding risk** |  |  |  |  |
| **progression or death** | **830 per 1000** | **695 per 1000** (642 to 744) | **HR 0.67**  (0.58 to 0.77) | 1381 (2 studies) | ⊕⊕⊕⊝ **moderate** |  |
| **OS** | **352 per 1000** | **346 per 1000** (305 to 998) | **HR 0.98**  (0.84 to 15) | 1381 (2 studies) | ⊕⊕⊝⊝ **low** |  |
| **CR** | **118 per 1000** | **139 per 1000** (106 to 184) | **RR 1.18**  (0.90 to 1.56) | 1381 (2 studies) | ⊕⊕⊝⊝ **low** |  |
| **ORR** | **722 per 1000** | **758 per 1000** (707 to 801) | **RR 1.05**  (0.98 to 1.11) | 1381 (2 studies) | ⊕⊕⊝⊝ **low** |  |
| **VGPR or CR** | **405 per 1000** | **413 per 1000** (364 to 466) | **RR 1.02**  (0.90 to 1.15) | 1381 (2 studies) | ⊕⊕⊝⊝ **low** |  |
| **neutropenia_Gr3,4** | **416 per 1000** | **437 per 1000** (396 to 483) | **RR 1.05**  (0.95 to 1.16) | 1381 (2 studies) | ⊕⊕⊝⊝ **low** |  |
| **anemia_Gr3,4** | **186 per 1000** | **199 per 1000** (162 to 247) | **RR 1.07**  (0.87 to 1.33) | 1381 (2 studies) | ⊕⊕⊝⊝ **low** |  |
| **thrombocytopenia_Gr3,4** | **173 per 1000** | **166 per 1000** (135 to 204) | **RR 0.96**  (0.78 to 1.18) | 1381 (2 studies) | ⊕⊕⊝⊝ **low** |  |
| **febrile neutropenia_Gr3,4** | **26 per 1000** | **66 per 1000** (21 to 205) | **RR 2.52**  (0.81 to 7.85) | 305 (1 study) | ⊕⊝⊝⊝ **very low** |  |
| **infection_Gr3,4** | **203 per 1000** | **246 per 1000** (203 to 299) | **RR 1.21**  (1.00 to 1.47) | 1381 (2 studies) | ⊕⊕⊝⊝ **low** |  |
| **DVT_Gr3,4** | **39 per 1000** | **13 per 1000** (3 to 64) | **RR 0.34**  (0.07 to 1.64) | 305 (1 study) | ⊕⊝⊝⊝ **very low** |  |
| *The basis for the assumed risk (e.g. the median control group risk across studies) is provided in footnotes. The corresponding risk (and its 95% confidence interval) is based on the assumed risk in the comparison group and the relative effect of the intervention (and its 95% CI). CI, confidence interval; RR, risk ratio; HR, hazard ratio; OS, overall survival; CR, complete response; ORR, overall response rate; VGPR, very good partioal response; Gr, grade; DVT, deep vein thrombosis | | | | | | |

**Figure 2. Results of meta-analysis**

**A) A meta-analysis of survival rates based on the continuous therapy**


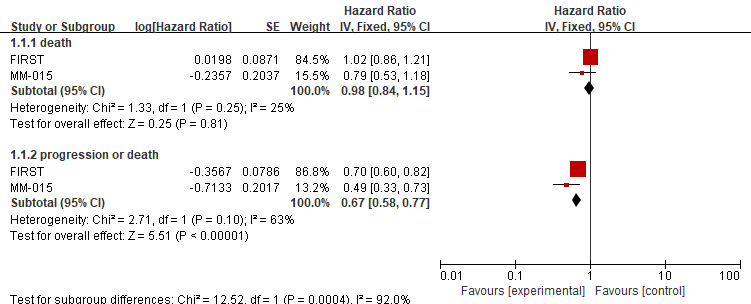


**B) A meta-analysis of response rates based on the continuous therapy**


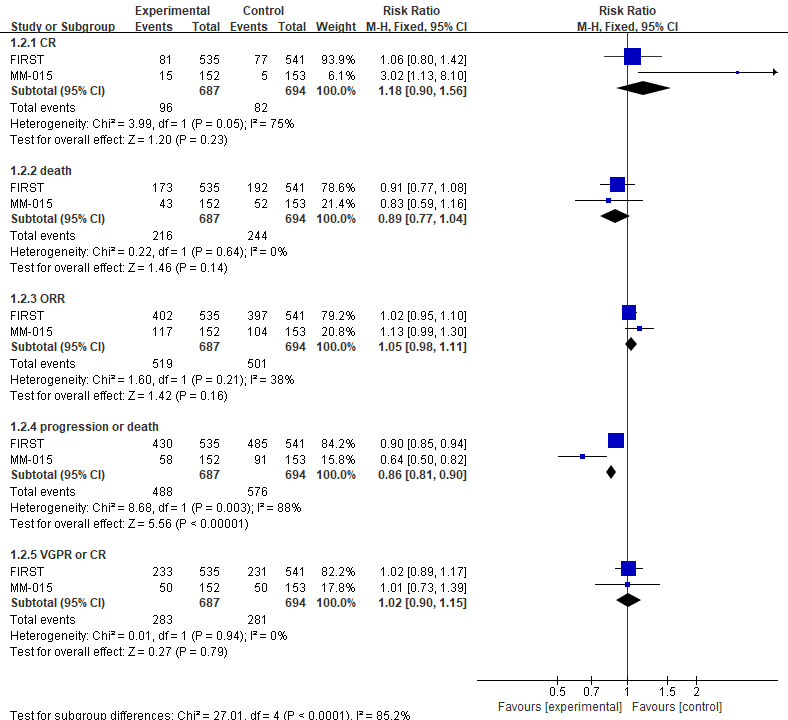


**C) A meta-analysis of progression-free survival based on the continuous therapy over different time points**


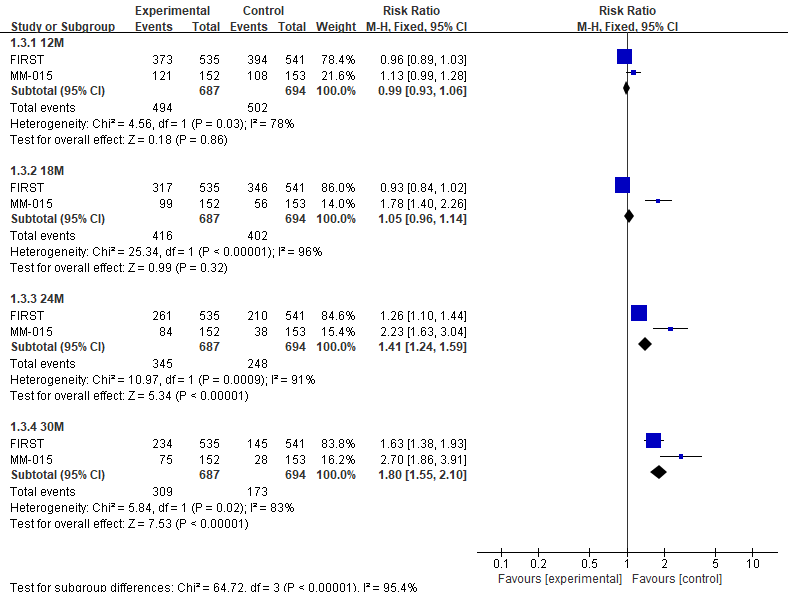


**D) A meta-analysis of overall survival based on the continuous therapy over different time points**


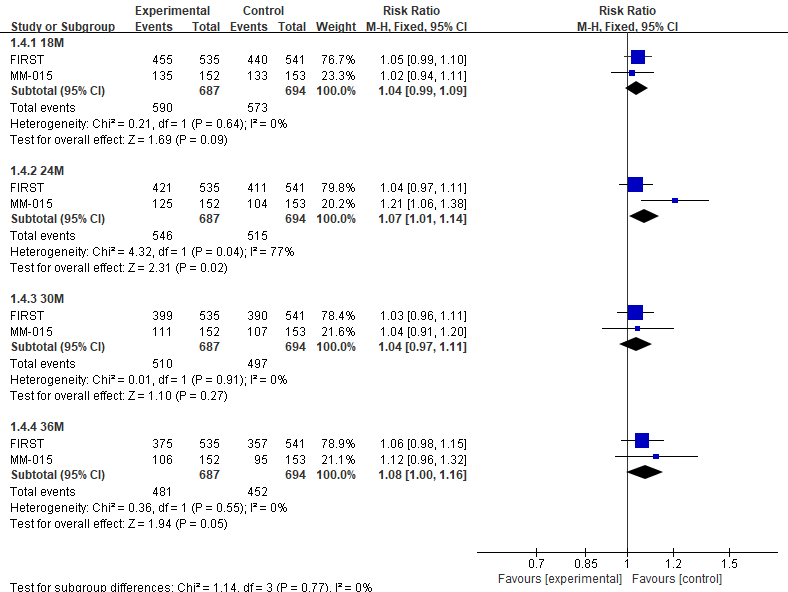
(


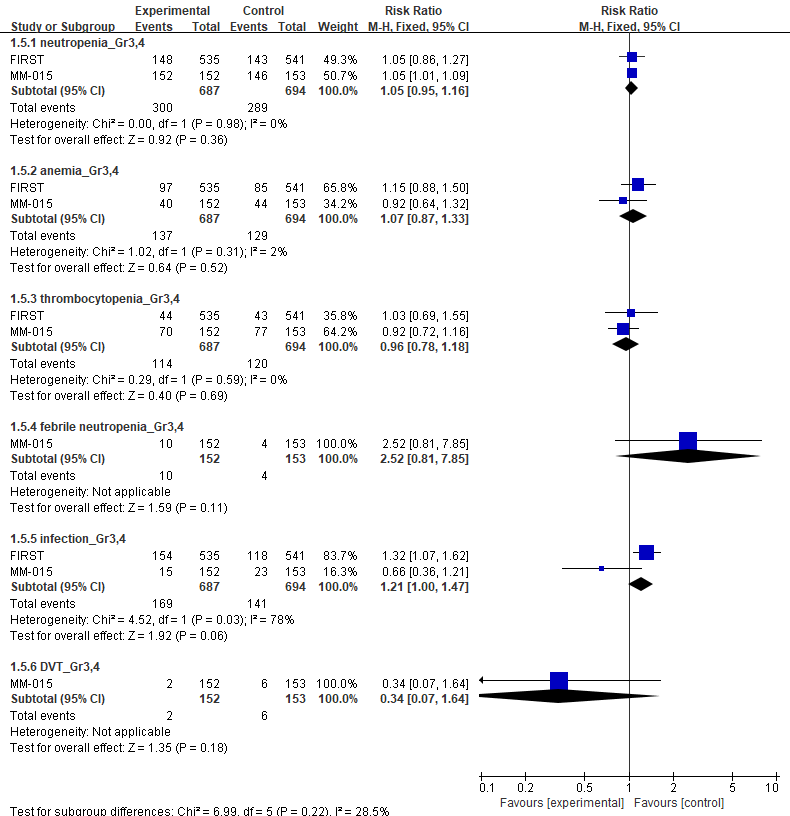
**E) A meta-analysis of adverse events based on the continuous therapy**

**Key Question 3. Is upfront autologous stem cell transplantation more effective than delayed autologous stem cell transplantation in transplant-eligible multiple myeloma?**

**Table 3. Summary of Findings table**

| **Outcomes** | **Illustrative comparative risks* (95% CI)** | | **Relative effect (95% CI)** | **No of Participants (studies)** | **SoE (GRADE)** |
| --- | --- | --- | --- | --- | --- |
|  | Assumed risk | Corresponding  risk |  |  |  |
| **OS FU: 5 yrs** | 181  per 1000 | 134 per 1000  (66 to 264) | HR 0.72  (0.34 to 1.53) | 3,044 (2 studies) | ⊕⊝⊝⊝ Very low |
| **PFS FU: 5 yrs** | 790  per 1000 | 722 per 1000  (505 to 903) | HR 0.82  (0.45 to 1.49) | 3044 (2 studies) | ⊕⊝⊝⊝ Very low |
| *The basis for the assumed risk (e.g. the median control group risk across studies) is provided in footnotes. The corresponding risk (and its 95% confidence interval) is based on the assumed risk in the comparison group and the relative effect of the intervention (and its 95% CI).  OS, overall survival; PFS, progression-free survival; FU, follow-up; CI, confidence interval; HR, hazard ratio | | | | | |

**Figure3. Result of meta-analysis**


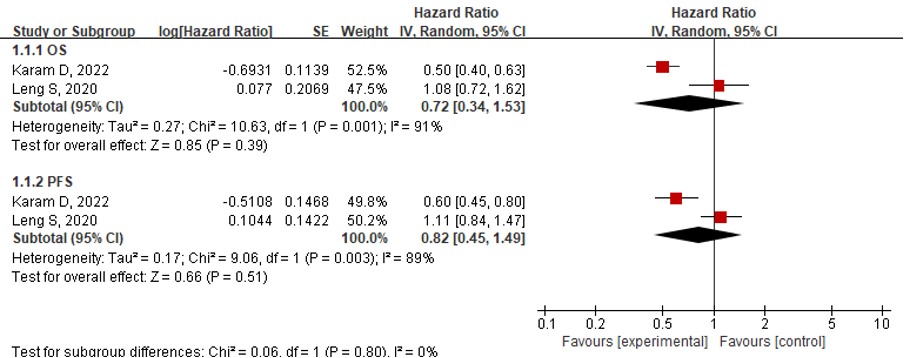


**Key Question 4.** **Dose tandem transplantation improve overall survival compared with a single autologous stem cell transplantation?**

**Table 4. Summary of Findings table**

| **Outcomes** | **Illustrative comparative risks***  **(95% CI)** | | **Relative effect (95% CI)** | **No of Participants (studies)** | **Quality of the evidence (GRADE)** | **Comments** |
| --- | --- | --- | --- | --- | --- | --- |
|  | Assumed  risk | Corresponding  risk |  |  |  |  |
| **OS** | **556 per 1000** | **589 per 1000** (506 to 678) | **RR 1.06**  (0.91 to 1.22) | 1913 (3 studies) | ⊕⊝⊝⊝ **very low** |  |
| **RFS** | **421 per 1000** | **509 per 1000** (446 to 581) | **RR 1.21**  (1.06 to 1.38) | 1322 (2 studies) | ⊕⊕⊝⊝ **low** |  |
| **CR+VGPR** | **364 per 1000** | **437 per 1000** (386 to 491) | **RR 1.20**  (1.06 to 1.35) | 1494 (3 studies) | ⊕⊕⊝⊝ **low** |  |
| **EFS** | **262 per 1000** | **383 per 1000** (310 to 472) | **RR 1.46**  (1.18 to 1.8) | 720 (2 studies) | ⊕⊕⊕⊝ **moderate** |  |
| **treatment related deaths** | **36 per 1000** | **50 per 1000** (25 to 101) | **RR 1.40**  (0.69 to 2.81) | 720 (2 studies) | ⊕⊕⊝⊝ **low** |  |
| **mucositis(Gr3/4)** | **245 per 1000** | **277 per 1000** (194 to 402) | **RR 1.13**  (0.79 to 1.64) | 321 (1 study) | ⊕⊕⊝⊝ **low** |  |
| **infection(Gr3/4)** | **209 per 1000** | **240 per 1000** (161 to 361) | **RR 1.15**  (0.77 to 1.73) | 321 (1 study) | ⊕⊕⊝⊝ **low** |  |
| **≥Gr3 non-hematologic toxicities** | **504 per 1000** | **509 per 1000** (438 to 584) | **RR 1.01**  (0.87 to 1.16) | 761 (2 studies) | ⊕⊕⊝⊝ **low** |  |
| **Secondary malignancies** | **43 per 1000** | **57 per 1000** (27 to 123) | **RR 1.33**  (0.62 to 2.87) | 501 (1 study) | ⊕⊕⊝⊝ **low** |  |
| **PFS(High risk)** Follow-up: 5 years | **256 per 1000** | **356 per 1000** (241 to 523) | **RR 1.39**  (0.94 to 2.04) | 228 (2 studies) | ⊕⊝⊝⊝  **very low** |  |
| **17p deletion 5yrs PFS** | **91 per 1000** | **23 per 1000** (9 to 62) | **HR 0.24**  (0.09 to 0.67) | 40 (1 study) | ⊕⊝⊝⊝  **very low** |  |
| **17p deletion 5yrs OS** | **318 per 1000** | **109 per 1000** (34 to 334) | **HR 0.30**  (0.09 to 1.06) | 40 (1 study) | ⊕⊝⊝⊝  **very low** |  |
| *The basis for the assumed risk (e.g. the median control group risk across studies) is provided in footnotes. The corresponding risk (and its 95% confidence interval) is based on the assumed risk in the comparison group and the relative effect of the intervention (and its 95% CI). CI, confidence interval; RR, risk ratio; HR, hazard ratio; OS, overall survival; RFS, relapse free survival; CR, complete response; VGPR, very good partial response; EFS, event free survival; Gr, grade; PFS, progression free survival | | | | | | |

**Figure 4. Results of meta-analysis**

**A) A meta-analysis of response rate and survival rates**


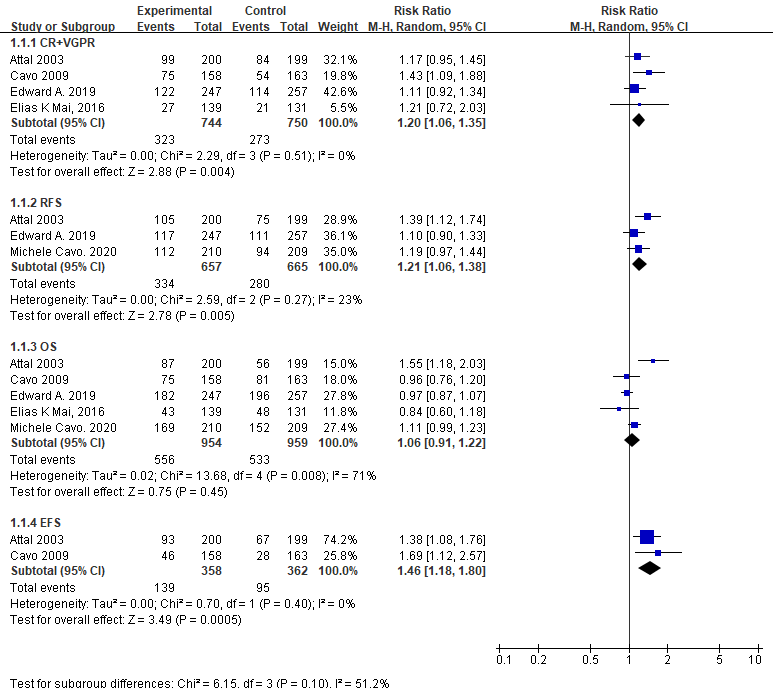


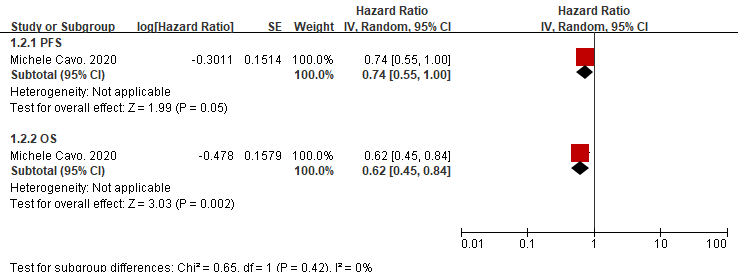


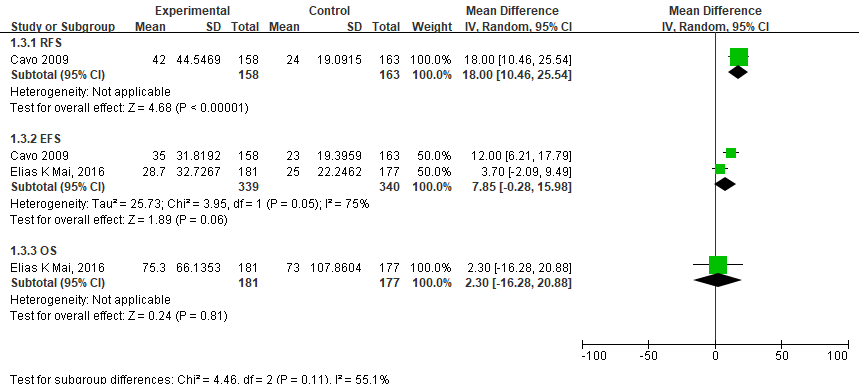


**B) A meta-analysis of survival rates in high-risk patients**


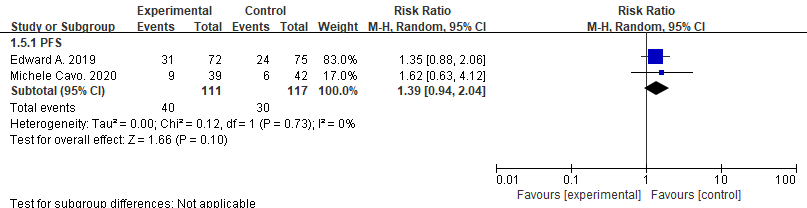


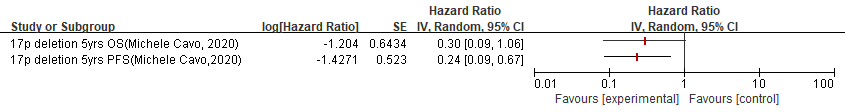


**C) A meta-analysis of adverse events**


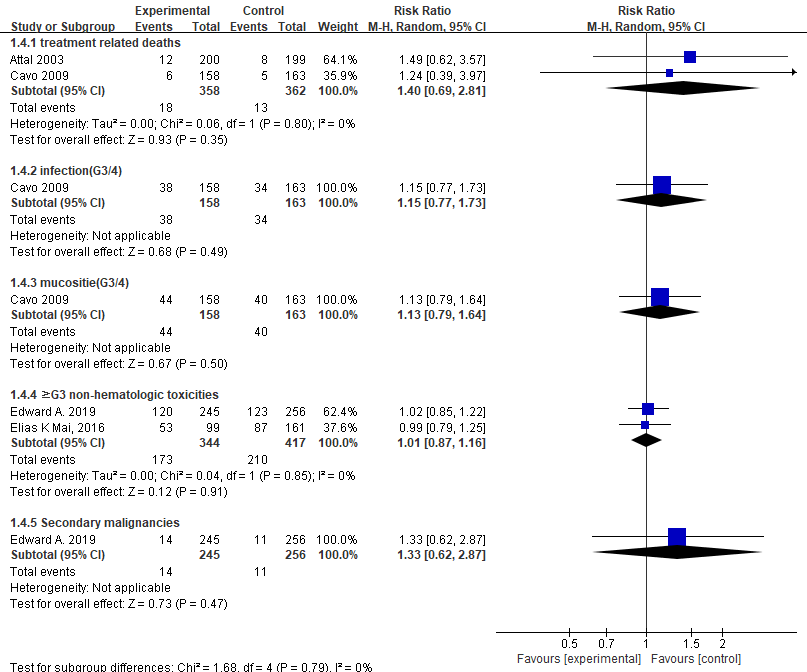


**Key Question 5. Does maintenance therapy after autologous stem cell transplantation improve survival in patients with multiple myeloma?**

**Table 5. Summary of Findings table**

| **Outcomes** | **Illustrative comparative risks* (95% CI)** | | **Relative effect (95% CI)** | **No of Participants (studies)** | **Quality of the evidence (GRADE)** | **Comments** |
| --- | --- | --- | --- | --- | --- | --- |
|  | Assumed risk | Corresponding risk |  |  |  |  |
| **OS** | **236 per 1000** | **179 per 1000** (152 to 209) | **HR 0.73**  (0.61 to 0.87) | 2445 (4 studies) | ⊕⊕⊕⊝ **moderate** |  |
| **PFS** | **509 per 1000** | **324 per 1000** (300 to 352) | **HR 0.55**  (0.50 to 0.61) | 3982 (6 studies) | ⊕⊕⊕⊕ **high** |  |
| **Febrile neutropenia** | **1 per 1000** | **5 per 1000** (1 to 47) | **RR 3.95**  (0.44 to 35.12) | 1492 (2 studies) | ⊕⊕⊝⊝ **low** |  |
| **Grade 3/4**  **neutropenia** | **148 per 1000** | **271 per 1000** (168 to 442) | **RR 1.83**  (1.13 to 2.98) | 2605 (4 studies) | ⊕⊕⊕⊕ **high** |  |
| **Grade 3/4**  **thrombocytopenia** | **28 per 1000** | **71 per 1000** (48 to 103) | **RR 2.56**  (1.75 to 3.75) | 2605 (4 studies) | ⊕⊕⊕⊝ **moderate** |  |
| **Infection** | **49 per 1000** | **91 per 1000** (59 to 139) | **RR 1.86**  (1.22 to 2.85) | 2145 (3 studies) | ⊕⊕⊕⊝ **moderate** |  |
| **Second primary cancer** | **32 per 1000** | **63 per 1000** (43 to 91) | **RR 1.94**  (1.34 to 2.81) | 2605 (4 studies) | ⊕⊕⊕⊝ **moderate** |  |
| *The basis for the assumed risk (e.g. the median control group risk across studies) is provided in footnotes. The corresponding risk (and its 95% confidence interval) is based on the assumed risk in the comparison group and the relative effect of the intervention (and its 95% CI). CI, confidence interval; RR, risk ratio; HR, hazard ratio; OS, overall survival; PFS, progression-free survival | | | | | | |

**Figure 5. Results of meta-analysis**

**A) A meta-analysis of progression-free survival**


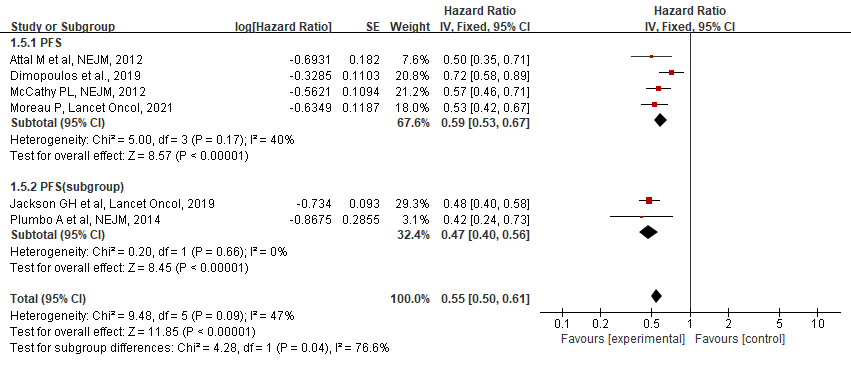


**B) A meta-analysis of overall survival**


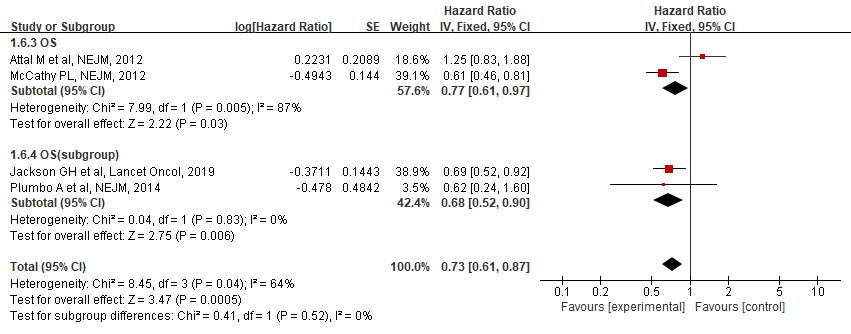


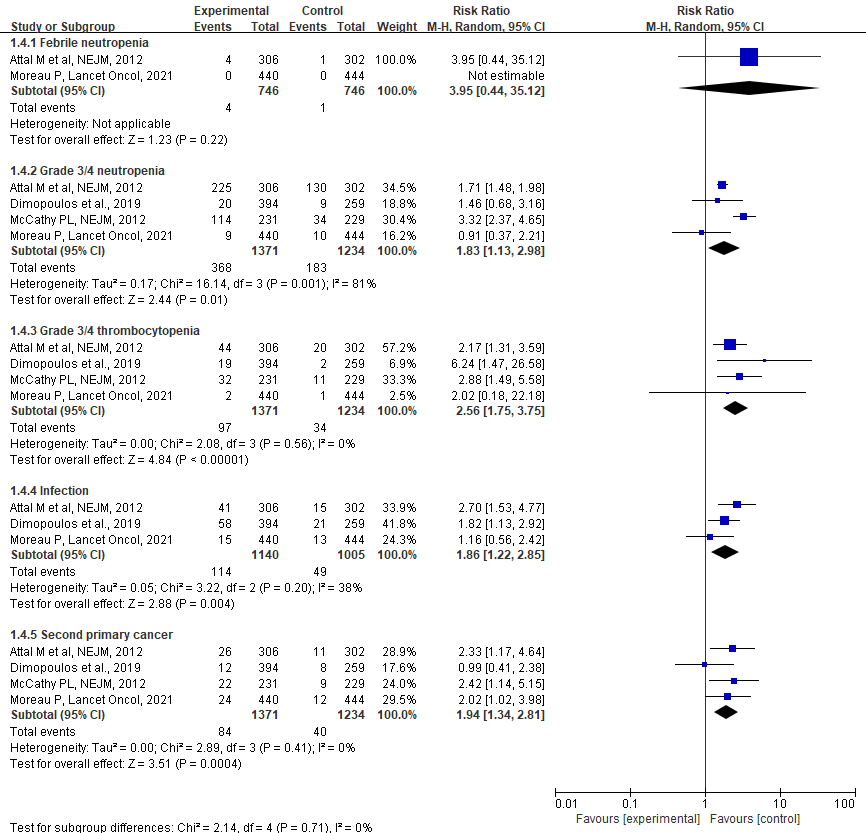
**C) A meta-analysis of adverse events**

**Key Question 6. In relapsed multiple myeloma, does treatment at the time of biochemical relapse improve survival compared to treatment at the time of symptomatic relapse?**

**Table 6. Summary of Findings table**

| **Biochemical compared to Clinical for** | | | | | | |
| --- | --- | --- | --- | --- | --- | --- |
| **Patient or population:** patients with  **Settings:**  **Intervention:** Biochemical **Comparison:** Clinical | | | | | | |
| **Outcomes** | **Illustrative comparative risks* (95% CI)** | | **Relative effect (95% CI)** | **No of Participants (studies)** | **Quality of the evidence (GRADE)** | **Comments** |
|  | Assumed risk | Corresponding risk |  |  |  |  |
|  | **Clinical** | **Biochemical** |  |  |  |  |
| **PFS** | **247 per 1000** | **145 per 1000** (142 to 180) | **HR 0.55**  (0.54 to 0.70) | 2808 (4 studies) | ⊕⊝⊝⊝ **very low** |  |
| **OS** | **648 per 1000** | **355 per 1000** (197 to 487) | **HR 0.42**  (0.21 to 0.64) | 3272 (6 studies) | ⊕⊝⊝⊝ **very low** |  |
| *The basis for the assumed risk (e.g. the median control group risk across studies) is provided in footnotes. The corresponding risk (and its 95% confidence interval) is based on the assumed risk in the comparison group and the relative effect of the intervention (and its 95% CI). CI, confidence interval; HR, hazard ratio; PFS, progression-free survival; OS, overall survival | | | | | | |

**Figure 6. Result of meta-analysis**

**
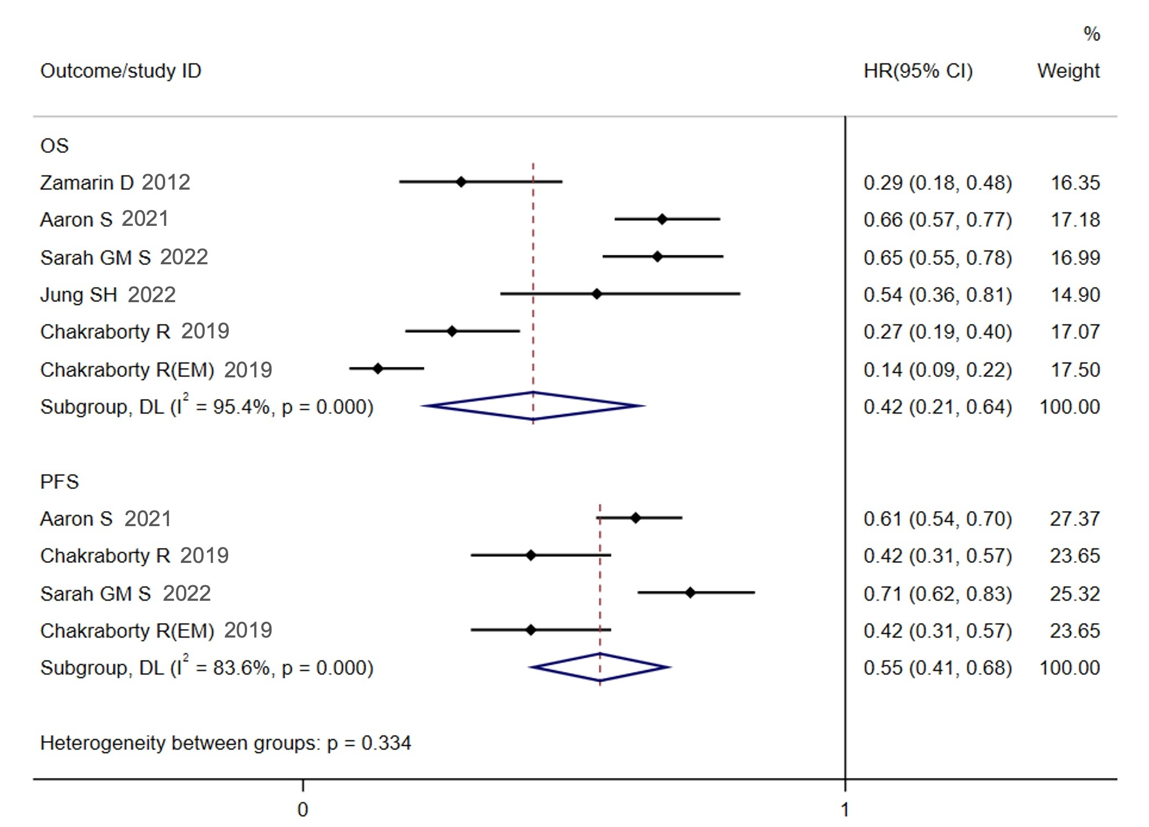
**

**Key Question 7. Is it effective to retreatment with previously effective agents in relapsed**

**multiple myeloma?**

**Table 7. Summary of Findings table**

| **Outcomes** | **Illustrative comparative risks* (95% CI)** | | **Relative effect (95% CI)** | **No of Participants (studies)** | **Quality of the evidence (GRADE)** | Comments |
| --- | --- | --- | --- | --- | --- | --- |
|  | Control proportion | Test proportion |  |  |  |  |
| **ORR** | 0.60(0.53-0.67) | 0.57(0.42-0.71) | Not estimable | 506 (10 studies) | ⊕⊝⊝⊝ **very low** |  |
| **CR** | 0.12(0.10-0.15) | 0.09(0.04-0.15) | Not estimable | 410 (9 studies) | ⊕⊝⊝⊝ **very low** |  |
| **VGPR** | 0.25(0.20-0.30) | 0.06(0.02-0.11) | Not estimable | 378 (8 studies) | ⊕⊝⊝⊝ **very low** |  |
| **PR** | 0.28(0.22-0.35) | 0.37(0.26-0.47) | Not estimable | 410 (9 studies) | ⊕⊝⊝⊝ **very low** |  |
| **median PFS** | - | 6.32(4.57-8.07) | Not estimable | 198 (4 studies) | ⊕⊝⊝⊝ **very low** |  |
| **median OS** | - | 14.35(8.82-19.88) | Not estimable | 233 (4 studies) | ⊕⊝⊝⊝ **very low** |  |
| **Neutropenia** | 0.23(0.17-0.28) | 0.05(-0.01-0.11) | Not estimable | 267 (3 studies) | ⊕⊝⊝⊝ **very low** |  |
| **Anemia** | 0.17(0.15-0.19) | 0.04(0.01-0.08) | Not estimable | 361 (4 studies) | ⊕⊝⊝⊝ **very low** |  |
| **Infection** | 0.11(0.07-0.15) | 0.01(-0.01-0.02) | Not estimable | 137 (2 studies) | ⊕⊝⊝⊝ **very low** |  |
| **Neutropathy** | 0.04(0.03-0.05) | 0.04(-0.01-0.08) | Not estimable | 276 (4 studies) | ⊕⊝⊝⊝ **very low** |  |
| **Pneumonia** | 0.10(0.09-0.11) | 0.05(0.02-0.08) | Not estimable | 226 (2 studies) | ⊕⊝⊝⊝ **very low** |  |
| **Thrombocytopenia** | 0.21(0.17-0.24) | 0.15(0.03-0.27) | Not estimable | 361 (4 studies) | ⊕⊝⊝⊝ **very low** |  |
| **Upper respiratory infection** | 0.01(0.01-0.01) | 0.01 | Not estimable | 96 (1 studies) | ⊕⊝⊝⊝ **very low** |  |
| **HTN** | 0.05(0.03-0.06) | 0.31 | Not estimable | 13 (1 studies) | ⊕⊝⊝⊝ **very low** |  |
| *The basis for the assumed risk (e.g. the median control group risk across studies) is provided in footnotes. The corresponding risk (and its 95% confidence interval) is based on the assumed risk in the comparison group and the relative effect of the intervention (and its 95% CI). CI, confidence interval; RR, risk ratio; ORR, overall response rate; CR, complete response; VGPR, very good partial response; PR, partial response; PFS, progression-free survival; OS, overall survival; HTN, hypertension | | | | | | |

**Figure 7. Results of meta-analysis**

**
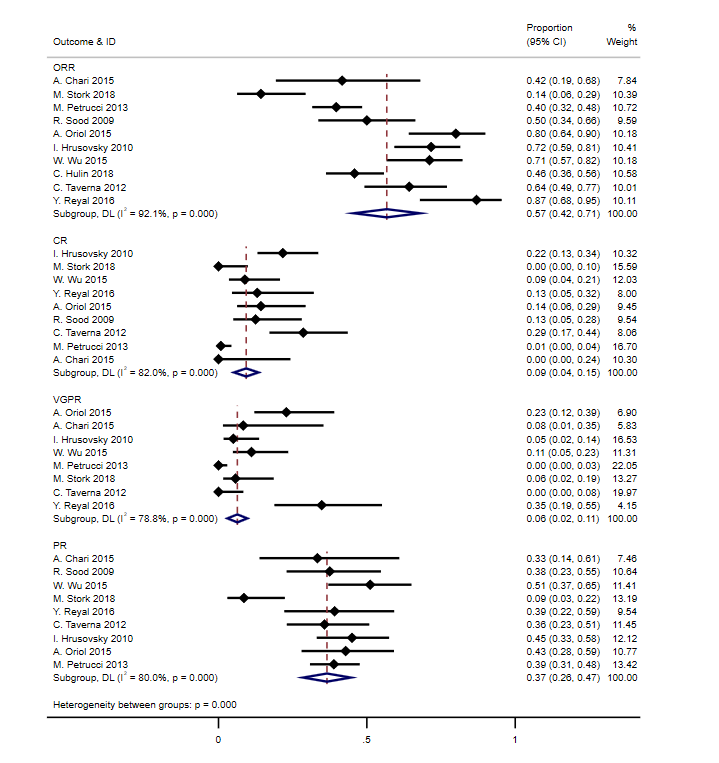
A) Response rates when treated with previous treatment.**

**B) Response rates when treated with agents other than those used in previous treatment**

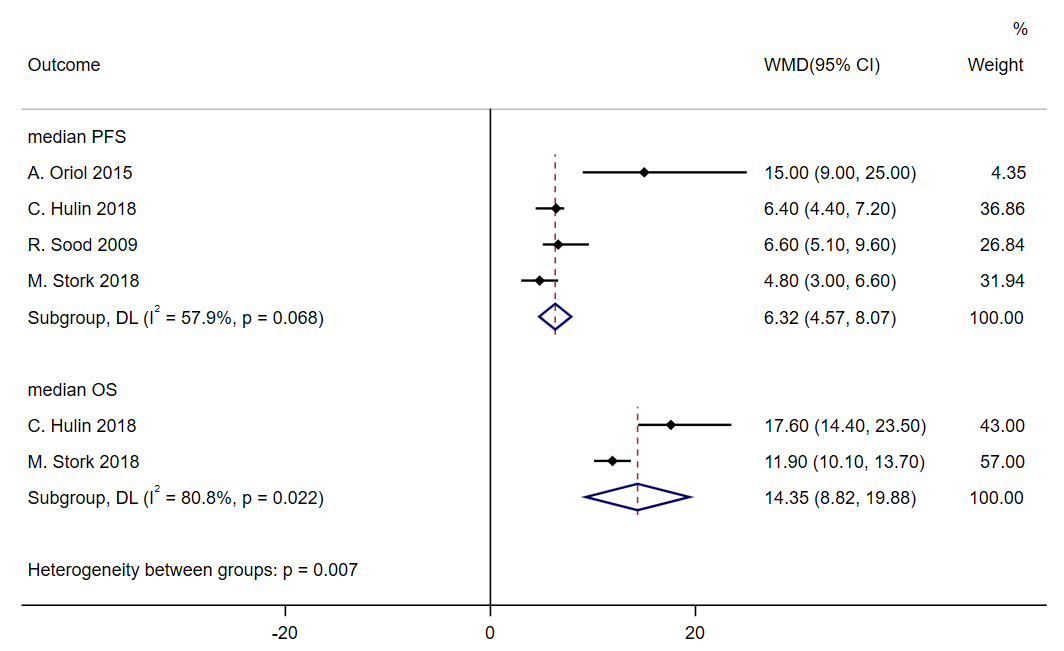
**C) Survival rates when treated with previous treatment.**

**D) Response rates in resue of bortezomib**


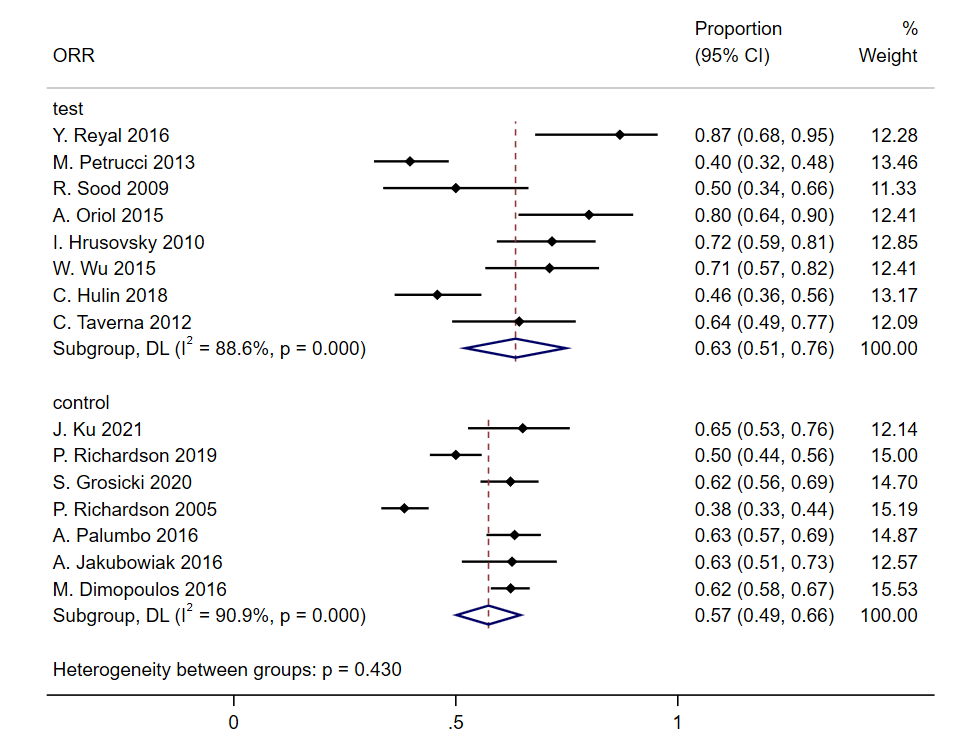


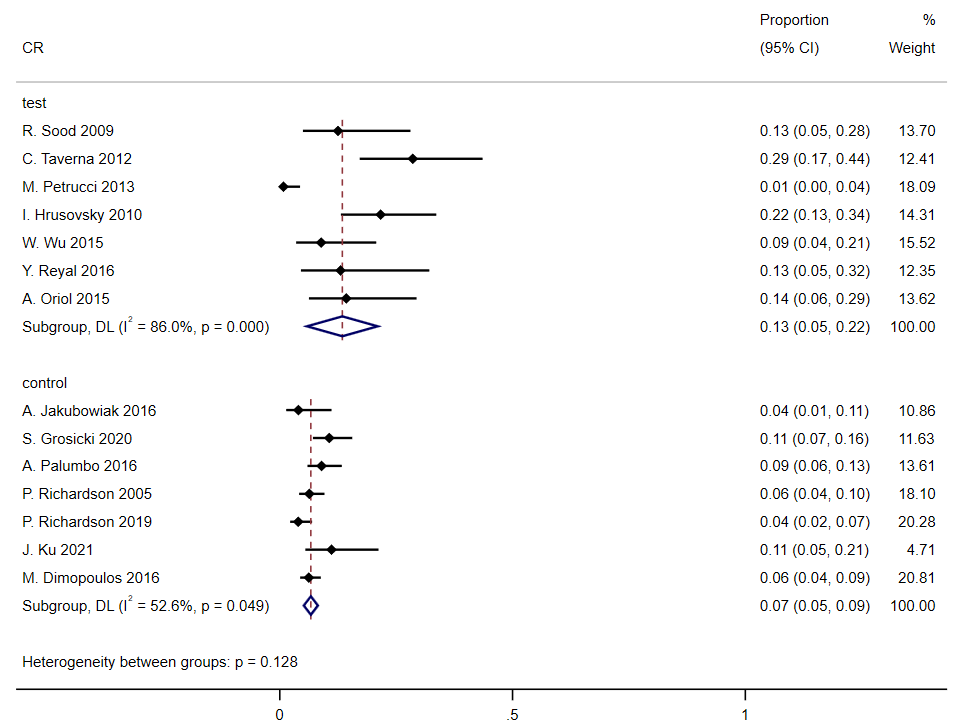


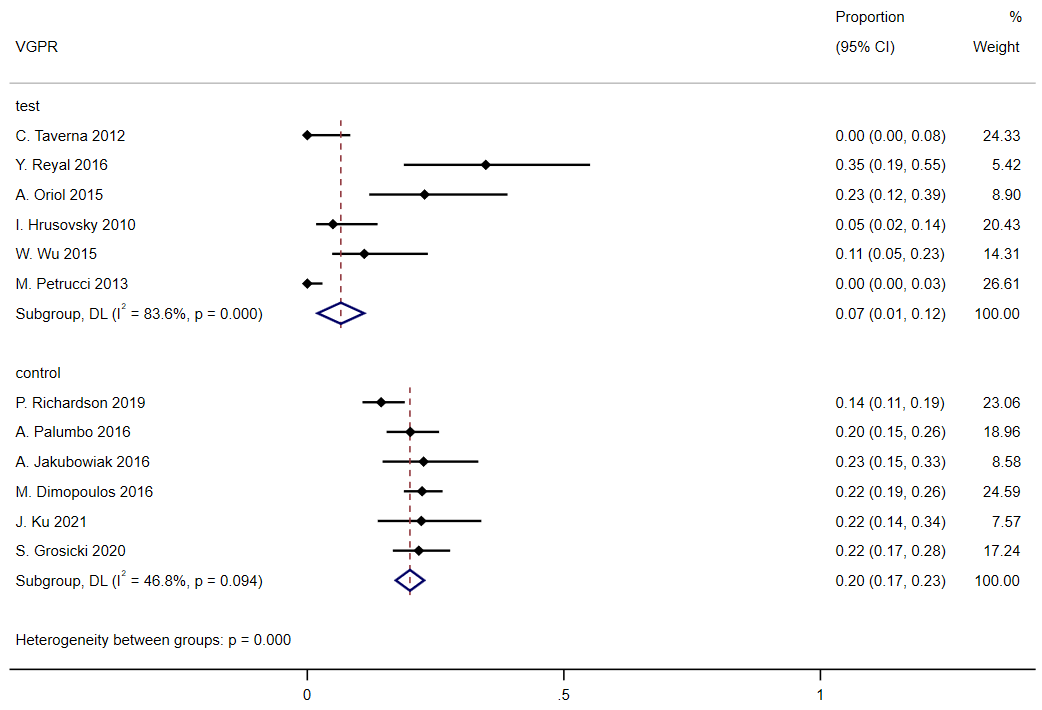


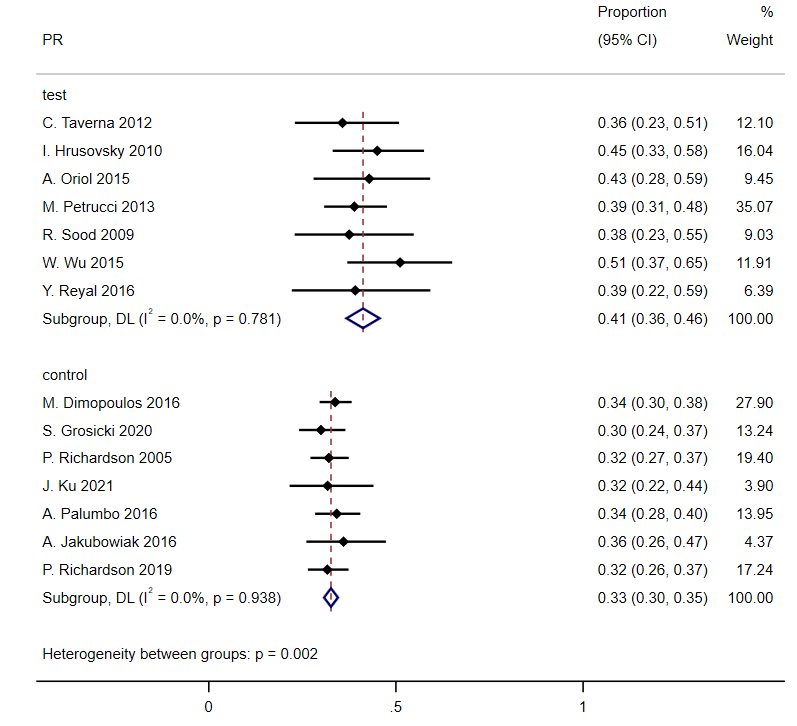


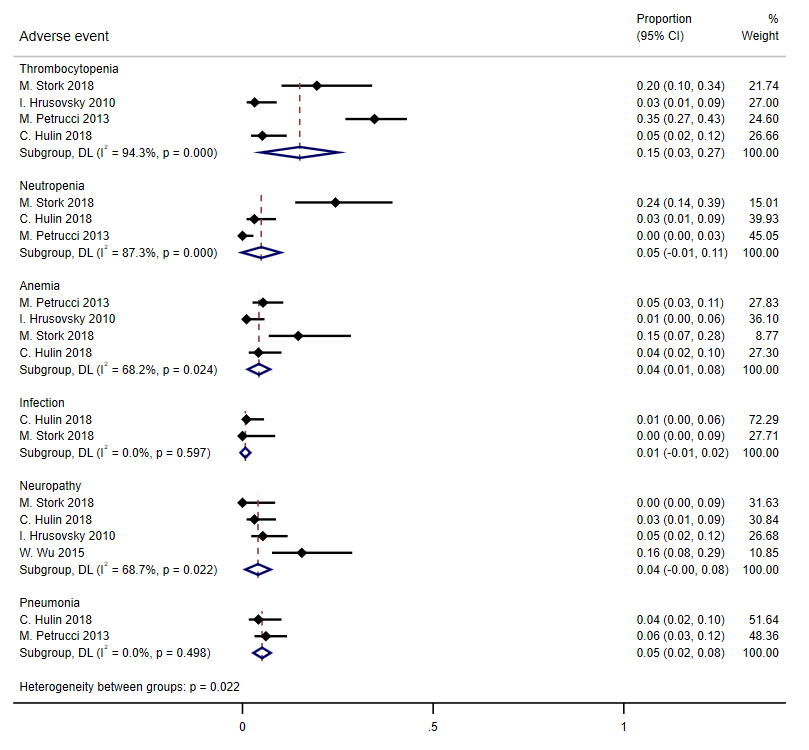
**E) A meta-analysis of adverse events**

**Key Question 8.** **Is the use of antibiotics or antivirals for infection prevention useful during initial induction therapy for newly diagnosed multiple myeloma?**

**Table 8. Summary of Findings table**

**A) Prophylactic antibiotic**


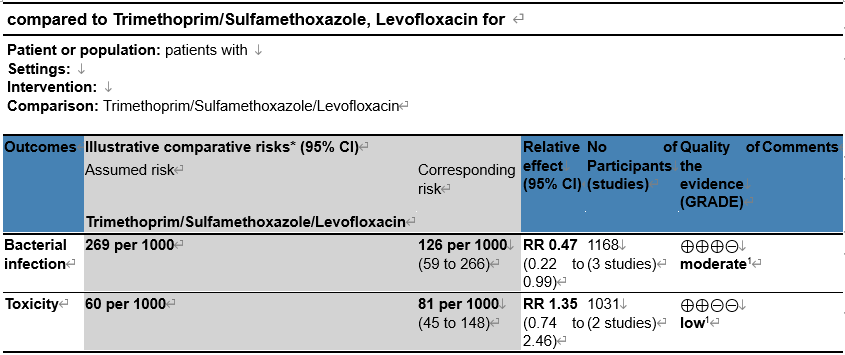


**B) Prophylactic antiviral agent**


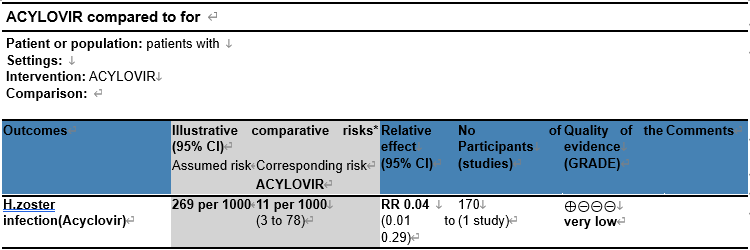


**Figure 8-1. Results of meta anaylsis of prophylactic antibiotic**

**A) A meta-analysis of efficacy of prophylactic antibiotics**


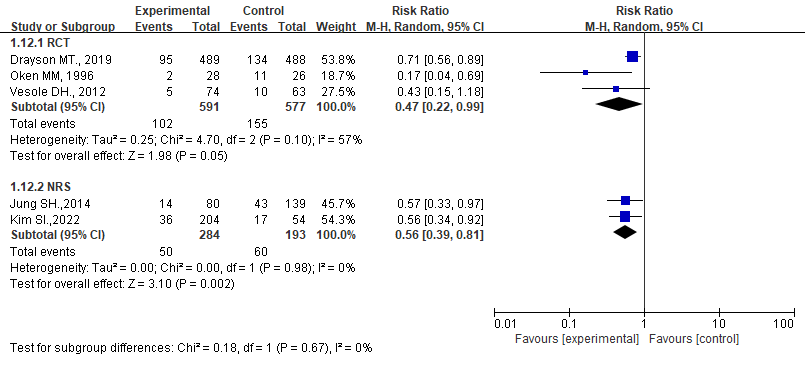


**B) A meta-analysis of adverse events**


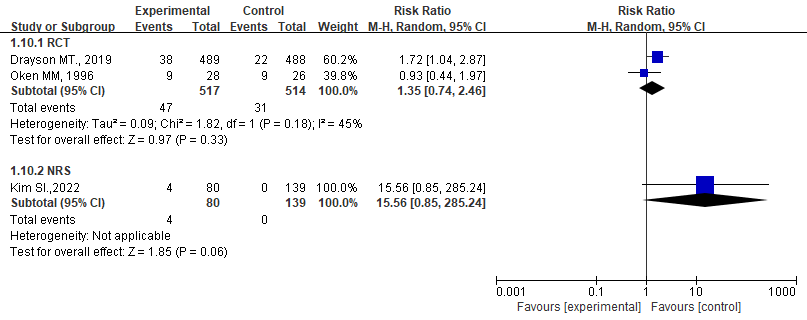


**Figure 8-2. A meta-analysis of efficacy of prophylactic antiviral agent**


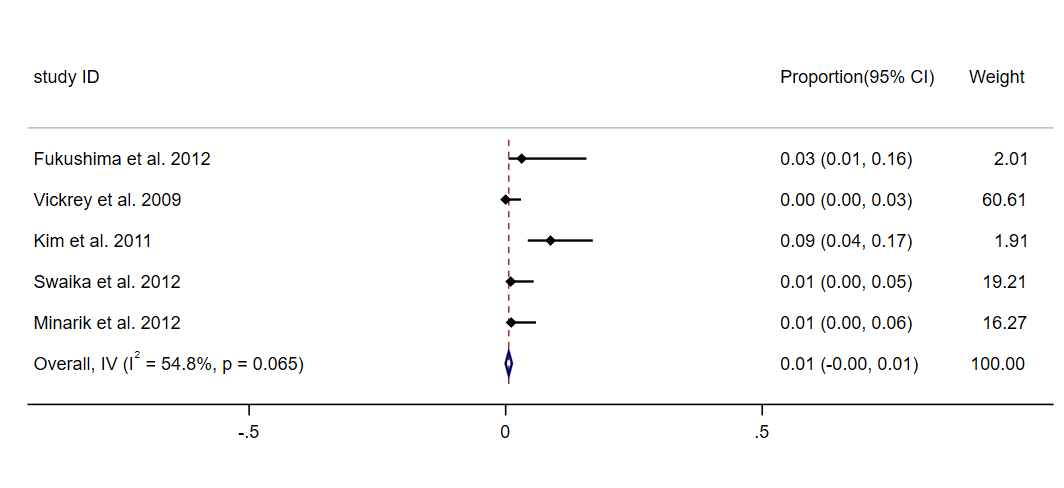

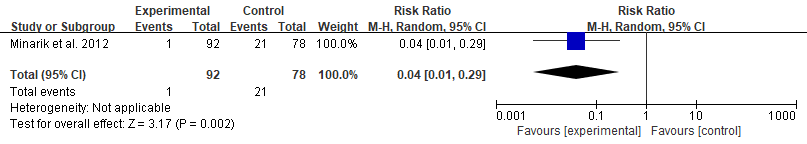


**Key Question 9.** **Is the use of bone resorption inhibitors effective in multiple myeloma?**

**Table 9. Table of Findings table**

**(A) Comparison of pamidronate or zoledronate with placebo or no treatment**

| **Outcomes** | **Illustrative comparative risks* (95% CI)** | | **Relative effect (95% CI)** | **No of Participants (studies)** | **Quality of the evidence (GRADE)** | **Comments** |
| --- | --- | --- | --- | --- | --- | --- |
|  | Assumed risk | Corresponding  risk |  |  |  |  |
| **OS** | **410 per 1000** | **286 per 1000** (236 to 330) | **HR 0.64**  (0.51 to 0.76) | 1479 (8 studies) | ⊕⊕⊕⊝ **moderate** |  |
| **PFS** | **477 per 1000** | **335 per 1000** (272 to 393) | **HR 0.63**  (0.49 to 0.77) | 982 (6 studies) | ⊕⊕⊕⊝ **moderate** |  |
| **Skeletal-related events (SREs)** | **320 per 1000** | **228 per 1000** (198 to 263) | **HR 0.67**  (0.57 to 0.79) | 1661 (7 studies) | ⊕⊕⊕⊝ **moderate** |  |
| **Osteonecrosis of Jaw (ONJ)** | **0 per 1000** | **4 per 1000** (0 to 10) | **RR 4.61** (0.99 to 21.35) | 1284 (6 studies) | ⊕⊕⊝⊝ **low** |  |
| **Gastrointestinal toxicity Grade III or IV** | **99 per 1000** | **8 per 1000** (89 to 184) | **RR 1.30**  (0.90 to 1.87) | 879 (3 studies) | ⊕⊕⊝⊝ **low** |  |
| *The basis for the assumed risk (e.g. the median control group risk across studies) is provided in footnotes. The corresponding risk (and its 95% confidence interval) is based on the assumed risk in the comparison group and the relative effect of the intervention (and its 95% CI). CI, confidence interval; RR, risk ratio; HR, hazard ratio; OS, overall survival; PFS, progression free survival; | | | | | | |

**(B) Comparison of Denosumab and Zoledronate**

| **Outcomes** | **Illustrative comparative risks***  **(95% CI)** | | **Relative effect (95% CI)** | **No of Participants (studies)** | **Quality of the evidence (GRADE)** | **Comments** |
| --- | --- | --- | --- | --- | --- | --- |
|  | Assumed risk | Corresponding risk |  |  |  |  |
|  | **Zoledronate** | **Denosumab** |  |  |  |  |
| **SRE_s** | **446 per 1000** | **439 per 1000** (395 to 490) | **HR 0.98**  (0.85 to 1.14) | 1718 (1 study) | ⊕⊕⊝⊝ **low** |  |
| **OS** | **150 per 1000** | **136 per 1000** (108 to 172) | **HR 0.90**  (0.70 to 1.16) | 1718 (1 study) | ⊕⊕⊝⊝ **low** |  |
| **PFS** | **305 per 1000** | **258 per 1000** (219 to 303) | **HR 0.82**  (0.68 to 0.99) | 1711 (1 study) | ⊕⊕⊝⊝ **low** |  |
| **ONJ** | **28 per 1000** | **41 per 1000** (25 to 69) | **RR 1.46**  (0.88 to 2.44) | 1702 (1 study) | ⊕⊕⊝⊝ **low** |  |
| **drug related**  **adverse events**  **Grade III or IV** | **58 per 1000** | **52 per 1000** (35 to 77) | **RR 0.90**  (0.61 to 1.34) | 1702 (1 study) | ⊕⊕⊝⊝ **low** |  |
| **Hypocalcemia** | **124 per 1000** | **169 per 1000** (134 to 214) | **RR 1.36**  (1.08 to 1.72) | 1702 (1 study) | ⊕⊕⊝⊝ **low** |  |
| **Renal dysfunction** | **46 per 1000** | **37 per 1000** (23 to 58) | **RR 0.80**  (0.50 to 1.26) | 1702 (1 study) | ⊕⊕⊝⊝ **low** |  |
| *The basis for the assumed risk (e.g. the median control group risk across studies) is provided in footnotes. The correspo nding risk (and its 95% confidence interval) is based on the assumed risk in the comparison group and the relative effect of the intervention (and its 95% CI). CI, confidence interval; RR, risk ratio; HR: hazard ratio; | | | | | | |

**Figure 9. Results of meta-analysis**

**A) Skeletal-related events**


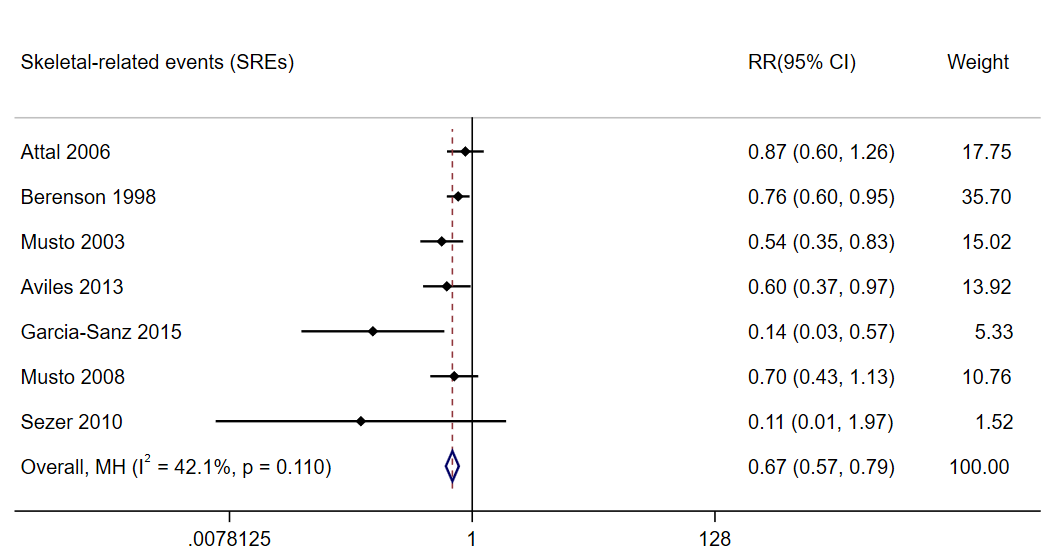


**B) Survival**


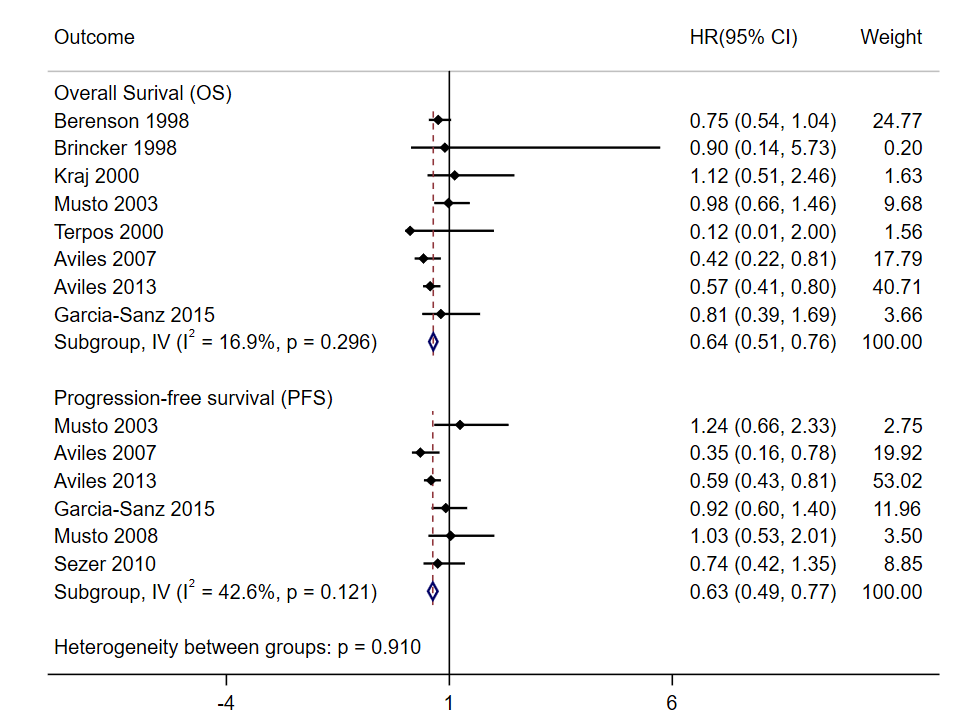


**C) Comparison of denosumab and zoledronate**


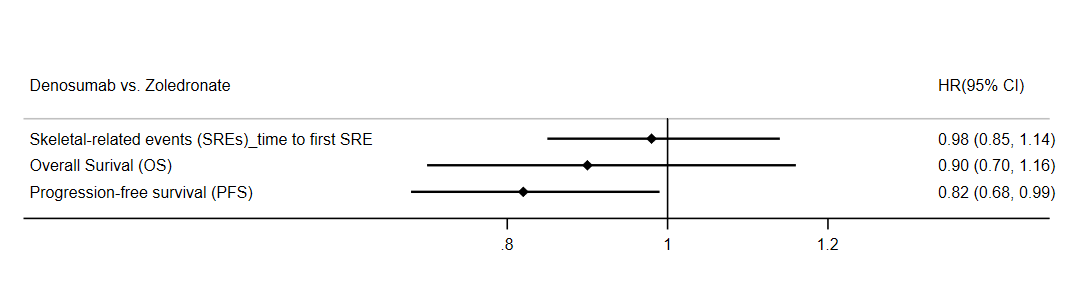


**D) Gatrointestinal toxicity Grade III or IV**


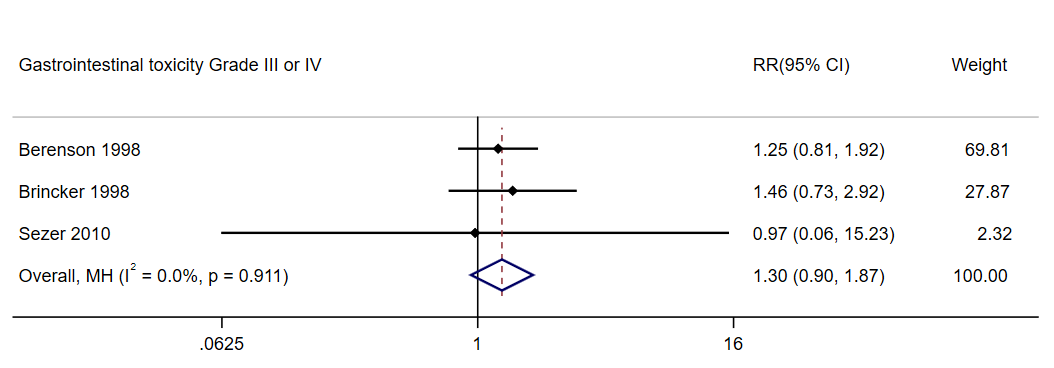


**E) Incidence of osteonecrosis of jaw**


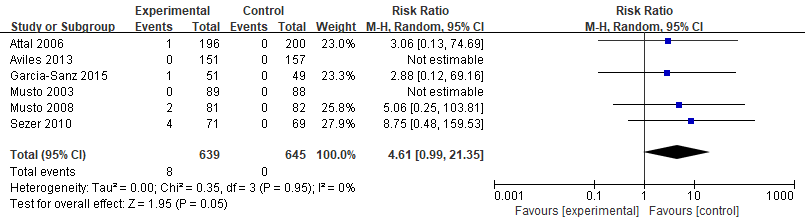


**F) Adverse events of denosumab and zoledronate**


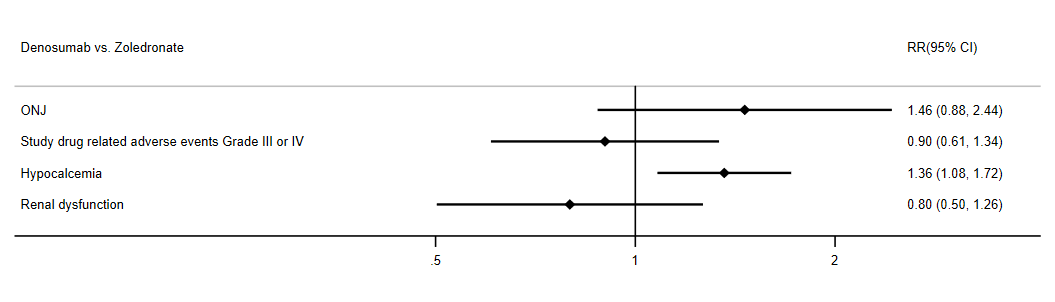


**Key Question 10. Is FDG PET/CT or MRI useful for prognosis prediction in newly diagnosed multiple myeloma?**

**Tablke 10-1 Summary of clinical studies**

**A) Clinical studies of FDG PET/CT**

| **Study** | **Year** | **Country** | **Design** | **Population** | **Median age (y)** | **Median f/u (m)** | **PET/CT parameters** | **Outcomes** |
| --- | --- | --- | --- | --- | --- | --- | --- | --- |
| Zamagni (90) | 2015 | Italy | Retro | 282 | 59 | 67 | FL | PFS, OS |
| McDonald (91) | 2017 | USA | Retro | 192 | NA | 101.52 | FL | PFS, OS |
| Davies (92) | 2018 | USA | Retro | 596 | 60.6 | 61.2 | FL | PFS, OS |
| Jung (93) | 2019 | Korea | Retro | 167 | 67 | 24.4 | FL | PFS, OS |
| Moon (94) | 2018 | Korea | Retro | 76 | 60.5 | 27.6 | FL | PFS, OS |
| Aljama (95) | 2018 | USA | Retro | 313 | 66 | 67 | FL | PFS, OS |
| Abe (96) | 2019 | Japan | Retro | 163 | 74.1 | 27.5 | FL | PFS, OS |
| Cho (97) | 2022 | Korea | Retro | 210 | 67 | 18.5 | FL | PFS, OS |
| Cho (98) | 2021 | Korea | Retro | 380 | 66 | 26 | FL | PFS, OS |
| Sachpekidis (99) | 2021 | Germany | Retro | 47 | 59.9 | 85.1 | FL | PFS |
| Rasche (100) | 2018 | USA | - | 404 | NA | 62.4 | FL | PFS |
| Fonti (101) | 2015 | Italy | Retro | 27 | 62 | 66 | FL | PFS |

**B) Clinical studies of MRI**

| **Study** | **Year** | **Country** | **Design** | **Population** | **Median age (y)** | **Median f/u (m)** | **MRI parameters** | **Outcomes** |
| --- | --- | --- | --- | --- | --- | --- | --- | --- |
| Lee (102) | 2022 | Korea | Retro | 214 | 67 | 37.9 | Diffuse | PFS, OS |
| Zhang (103) | 2021 | China | Pros | 102 | 60 | 50.2 | Diffuse | PFS, OS |
| Mai (104) | 2015 | Germany | Retro | 161 | 58 | 64 | Diffuse, FL | PFS, OS |
| Song (105) | 2015 | Korea | Retro | 168 | 69 | 34.1 | Diffuse | PFS, OS |
| Song (106) | 2014 | Korea | Retro | 79 | 53.5 | 46.1 | Diffuse | PFS, OS |
| Rasche (100) | 2018 | USA | - | 404 | > 65 (35%) | 62.4 | FL | PFS, OS |
| Bartel (107) | 2009 | Italy | - | 239 | > 65 (28%) | 43 | FL | PFS, OS |
| Walker (108) | 2007 | USA | - | 420 | NA | 55 | FL | OS |
| Moulopoulos (109) | 2005 | Greece | Retro | 142 | ≥65, (30%) | NA | Diffuse | OS |
| Moulopoulos (110) | 2012 | Greece | Retro | 134 | 67 | NA | Diffuse | OS |

**Table 10-2. Summary of Findings table**

**A) Comparison of survival based on FDG PET/CT and MRI**

| **Outcomes** | **Illustrative comparative risks* (95% CI)** | | **Relative effect (95% CI)** | **No of Participants (studies)** | **Quality of the evidence (GRADE)** | **Comments** |
| --- | --- | --- | --- | --- | --- | --- |
|  | Assumed risk | Corresponding risk |  |  |  |  |
| **PET/CT(PFS)** | **553 per 1000** | **830 per 1000** (646 to 919) | **HR 2.20**  (1.29 to 3.12) | 1142 (5 studies) | ⊕⊝⊝⊝ **very low** |  |
| **PET/CT(OS)** | **298 per 1000** | **501 per 1000** (320 to 633) | **HR 1.96**  (1.09 to 2.83) | 762 (4 studies) | ⊕⊝⊝⊝ **very low** |  |
| **MRI(PFS)_Diffuse** | **440 per 1000** | **730 per 1000** (648 to 792) | **HR 2.26**  (1.80 to 2.71) | 622 (4 studies) | ⊕⊝⊝⊝ **very low** |  |
| **MRI(PFS) _High FL** | **373 per 1000** | **489 per 1000** (143 to 696) | **HR 1.44**  (0.33 to 2.55) | 565 (2 studies) | ⊕⊝⊝⊝ **very low** |  |
| **MRI(OS)_Diffuse** | **374 per 1000** | **653 per 1000** (569 to 718) | **HR 2.26**  (1.80 to 2.71) | 677 (4 studies) | ⊕⊝⊝⊝ **very low** |  |
| **MRI(OS) _High FL** | **236 per 1000** | **324 per 1000** (34 to 525) | **HR 1.45**  (0.13 to 2.76) | 565 (2 studies) | ⊕⊝⊝⊝ **very low** |  |
| *The basis for the assumed risk (e.g. the median control group risk across studies) is provided in footnotes. The corresponding risk (and its 95% confidence interval) is based on the assumed risk in the comparison group and the relative effect of the intervention (and its 95% CI).  CI, confidence interval; H, hazard ratio; PFS, progression-free survival; OS, overall survival; FL, focal lesion | | | | | | |

**B) Sensitivity and Specificity of FDG PET/CT and MRI from the perspective of PFS**

**Should PET/CT vs. MRI be used to diagnose PFS in [health problem and/or population]?**

**Patient or population**: [health problem and/or population]

**Setting**:

**New test**: MRI |**Cut-off value**:

**Pooled sensitivity PET/CT**:0.56 (95% CI: 0.52 to 0.59)|**Pooled specificity PET/CT**:0.64 (95% CI: 0.60 to 0.68)

**Pooled sensitivity MRI**:0.83 (95% CI: 0.78 to 0.87)|**Pooled specificity MRI**:0.39 (95% CI: 0.31 to 0.46)

| **Test result** | **Number of results per 1,000 patients tested (95% CI)** | | | | **Number of participants  (studies)** | **Certainty of the Evidence (GRADE)** |
| --- | --- | --- | --- | --- | --- | --- |
|  | **Prevalence40%**  Typically seen in | | **Prevalence50%**  Typically seen in | |  |  |
|  | PET/CT | MRI | PET/CT | MRI |  |  |
| **True positives** | 224 (208 to 236) | 332 (312 to 348) | 280 (260 to 295) | 415 (390 to 435) | PET/CT  2499 (10)  MRI  724(5) | ⨁⨁◯◯ Low |
|  | **108 fewer TP in PET/CT** | | **135 fewer TP in PET/CT** | |  |  |
| **False negatives** | 176 (164 to 192) | 68 (52 to 88) | 220 (205 to 240) | 85 (65 to 110) |  |  |
|  | **108 more FN in PET/CT** | | **135 more FN in PET/CT** | |  |  |
| **True negatives** | 384 (360 to 408) | 234 (186 to 276) | 320 (300 to 340) | 195 (155 to 230) | PET/CT  2499 (10)  MRI  724(5) | ⨁◯◯◯ Very low |
|  | **150 more TN in PET/CT** | | **125 more TN in PET/CT** | |  |  |
| **False positives** | 216 (192 to 240) | 366 (324 to 414) | 180 (160 to 200) | 305 (270 to 345) |  |  |
|  | **150 fewer FP in PET/CT** | | **125 fewer FP in PET/CT** | |  |  |

**CI:** confidence interval

**C) Sensitivity and Specificity of FDG PET/CT and MRI from the perspective of OS**

**Should PET/CT vs. MRI be used to diagnose OS in [health problem and/or population]?**

**Patient or population**: [health problem and/or population]

**Setting**:

**New test**: MRI |**Cut-off value**:

**Pooled sensitivity PET/CT**:0.62 (95% CI: 0.57 to 0.67)|**Pooled specificity PET/CT**:0.63 (95% CI: 0.57 to 0.68)

**Pooled sensitivity MRI**:0.78 (95% CI: 0.65 to 0.88)|**Pooled specificity MRI**:0.42 (95% CI: 0.26 to 0.60)

| **Test result** | **Number of results per 1,000 patients tested (95% CI)** | | | | **Number of participants  (studies)** | **Certainty of the Evidence (GRADE)** |
| --- | --- | --- | --- | --- | --- | --- |
|  | **Prevalence30%**  Typically seen in | | **Prevalence40%**  Typically seen in | |  |  |
|  | PET/CT | MRI | PET/CT | MRI |  |  |
| **True positives** | 186 (171 to 201) | 234 (195 to 264) | 248 (228 to 268) | 312 (260 to 352) | PET/CT  2021 (7)  MRI  995(7) | ⨁◯◯◯ Very low |
|  | **48 fewer TP in PET/CT** | | **64 fewer TP in PET/CT** | |  |  |
| **False negatives** | 114 (99 to 129) | 66 (36 to 105) | 152 (132 to 172) | 88 (48 to 140) |  |  |
|  | **48 more FN in PET/CT** | | **64 more FN in PET/CT** | |  |  |
| **True negatives** | 441 (399 to 476) | 294 (182 to 420) | 378 (342 to 408) | 252 (156 to 360) | PET/CT  2021 (7)  MRI  995(7) | ⨁◯◯◯ Very low |
|  | **147 more TN in PET/CT** | | **126 more TN in PET/CT** | |  |  |
| **False positives** | 259 (224 to 301) | 406 (280 to 518) | 222 (192 to 258) | 348 (240 to 444) |  |  |
|  | **147 fewer FP in PET/CT** | | **126 fewer FP in PET/CT** | |  |  |

**CI:** confidence interval

**Figure 10. Results of meta-analysis**

**A) Progression-free survival**


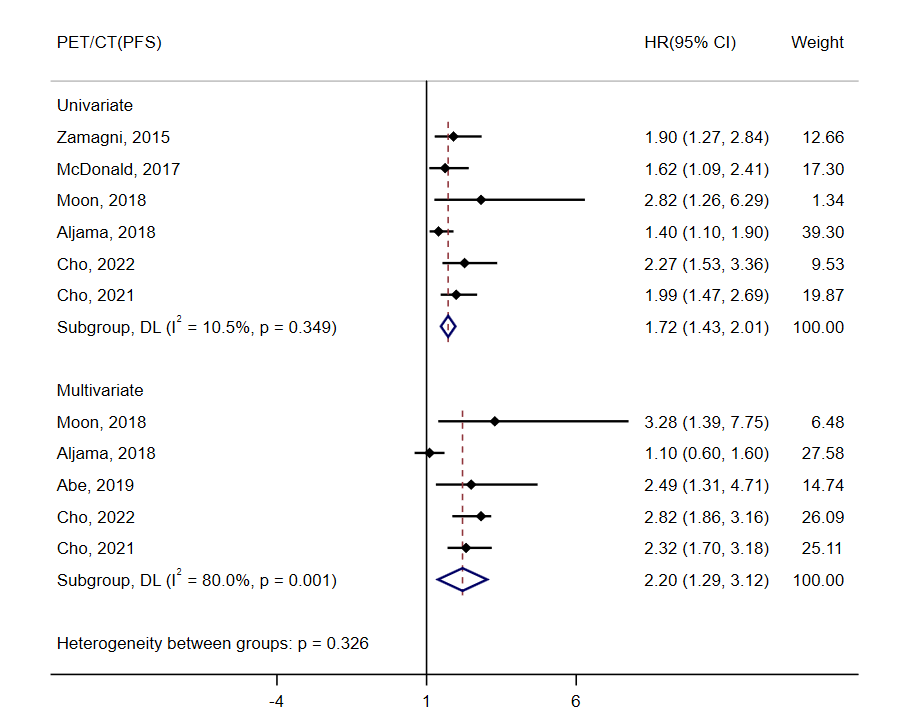

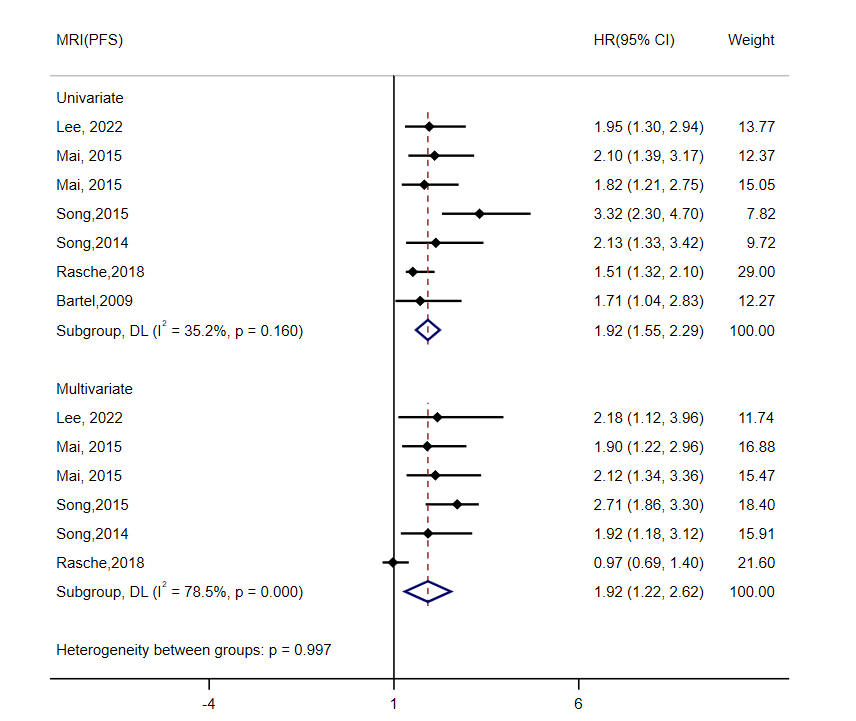


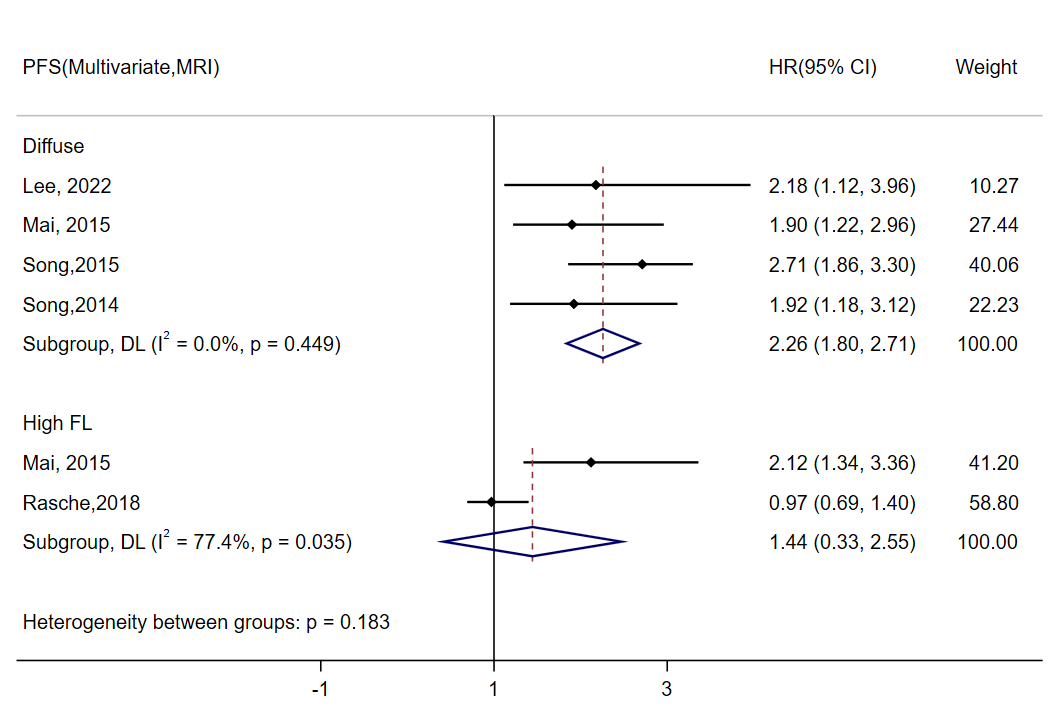

**B) Overall survival**


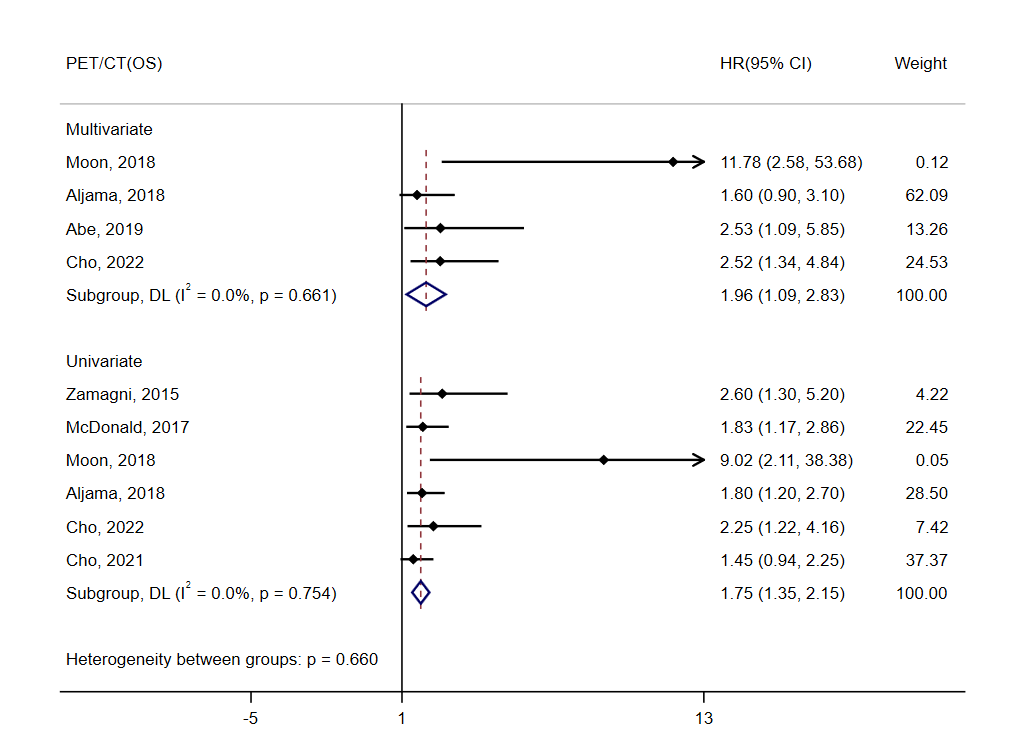

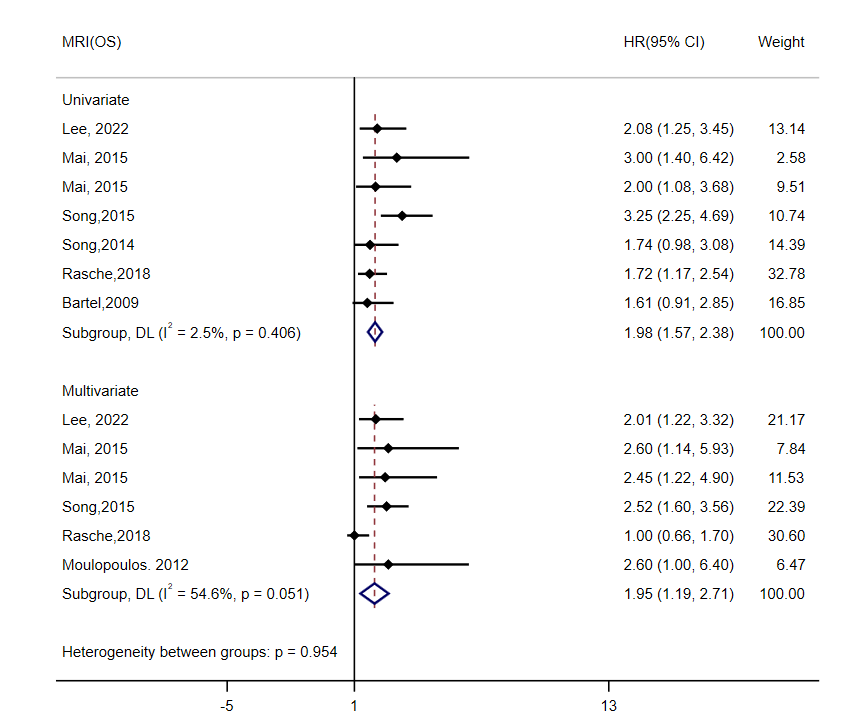


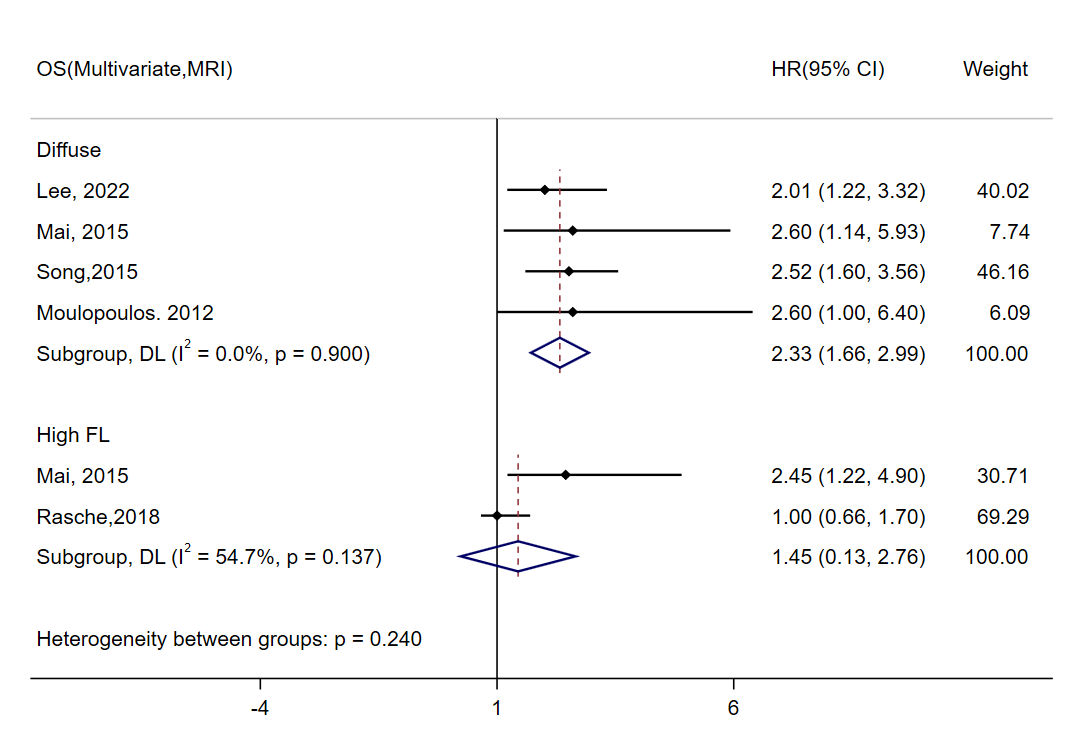

**Key Question 11.** **Is assessment of minimal residual disease useful in newly diagnosed multiple myeloma patients?**

**Table 11. Summary of Findings table**

| **MM MRD for** | | | | | | |
| --- | --- | --- | --- | --- | --- | --- |
| **Patient or population:** patients with newly diagnosed multiple myeloma  **Settings:** MM treatment- chemotherapy and autologous hematopoietic stem cell transplantation  **Intervention:** MM MRD method - multicolor flow cytometry /next-generation flow cytometry or  next-generation sequencing | | | | | | |
| **Outcomes** | **Illustrative comparative risks* (95% CI)** | | **Relative effect (95% CI)** | **No of Participants (studies)** | **Quality of the evidence (GRADE)** | **Comments** |
|  | Assumed risk | Corresponding risk |  |  |  |  |
|  | **Control** | **MMMRD** |  |  |  |  |
| **OS** | **286 per 1000** | **114 per 1000** (96 to 135) | **HR 0.36**  (0.3 to 0.43) | 3973 (14 studies) | ⊕⊝⊝⊝ **very low** |  |
| **PFS** | **416 per 1000** | **140 per 1000** (116 to 163) | **HR 0.28**  (0.23 to 0.33) | 9421 (29 studies) | ⊕⊝⊝⊝ **very low** |  |
| *The basis for the assumed risk (e.g. the median control group risk across studies) is provided in footnotes. The corresponding risk (and its 95% confidence interval) is based on the assumed risk in the comparison group and the relative effect of the intervention (and its 95% CI). CI, Confidence interval; HR, Hazard ratio; MM, multiple myeloma; MRD, minimal residual disease; OS, overall survival; PFS, progression-free survival. | | | | | | |

**Figure 11. Results of meta-analysis**

**A) Meta-analysis of progression-free survival according to MRD negativity**


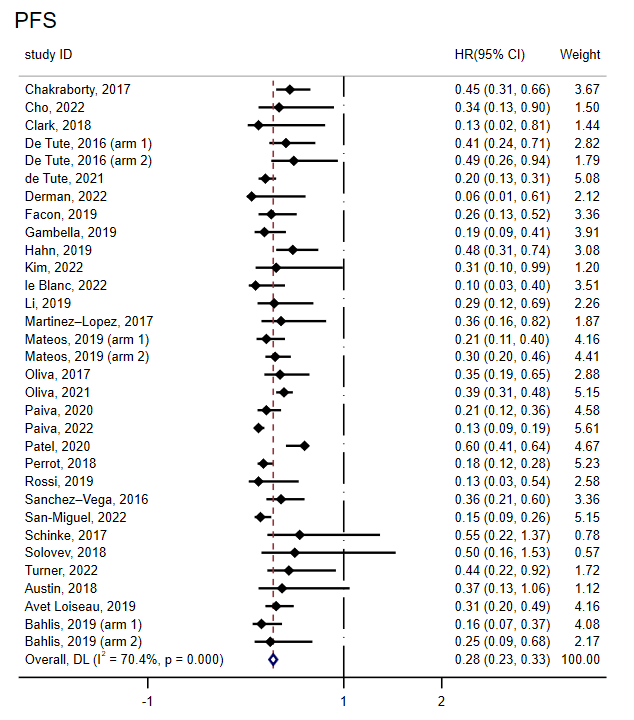


**B)** **Meta-analysis of progression-free survival according to MRD negativity; Sensitivity thresholds of 10^-4^(10^-4^ to 10^-5^) or 10^-5^(under 10^-5^)**

**
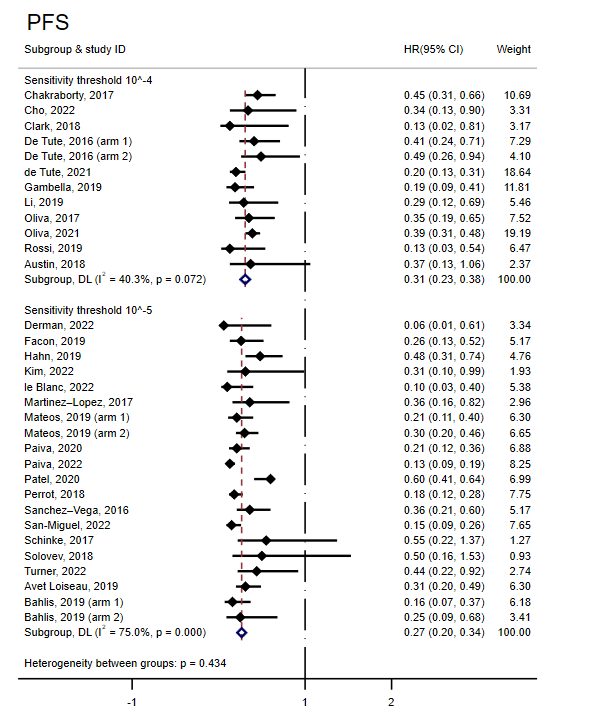
**

*10^-4^ means 10^-4^ to 10^-5^ sensitivity; 10^-5^ means under 10^-5^ sensitivity

**C)** **Meta-analysis of progression-free survival according to MRD negativity after ASCT or chemotherapy**


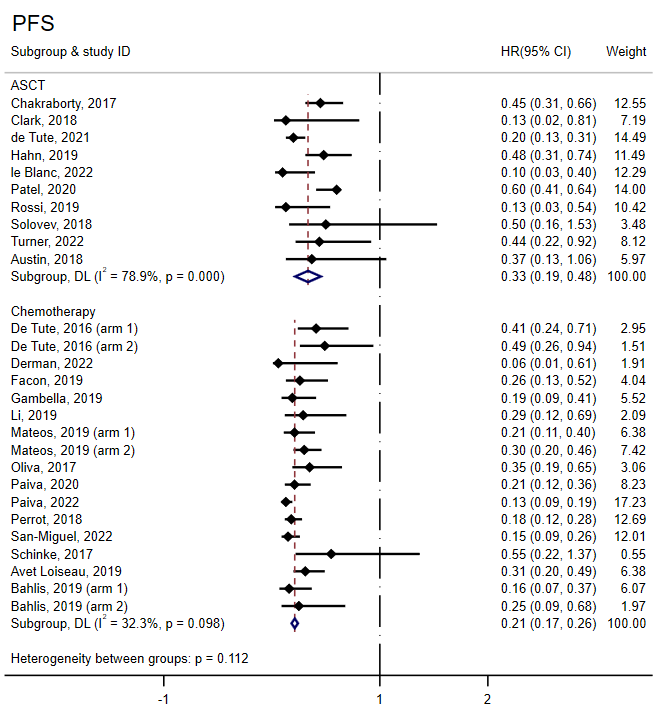


***ASCT,** autologous hematopoietic stem cell transplantation

**D)** **Meta-analysis of progression-free survival according to MRD negativity at different time points after treatment (under 6 months, 6-12 months, and over 12 months)**


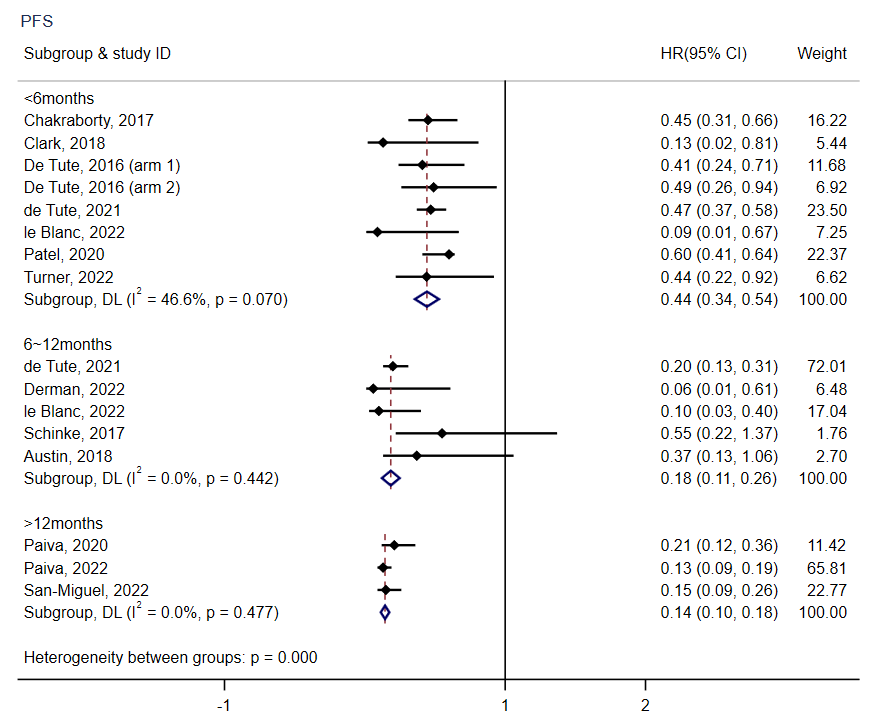


**E)** **Meta-analysis of overall survival according to MRD negativity**


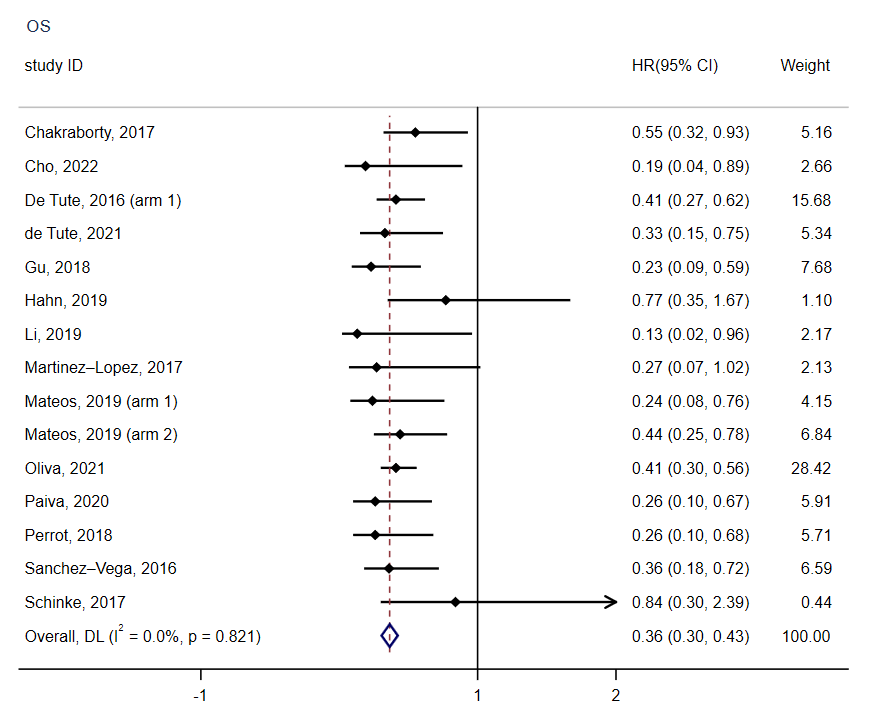


**F)** **Meta-analysis of overall survival according to MRD negativity; Sensitivity thresholds of 10^-4^ or 10^-5^**


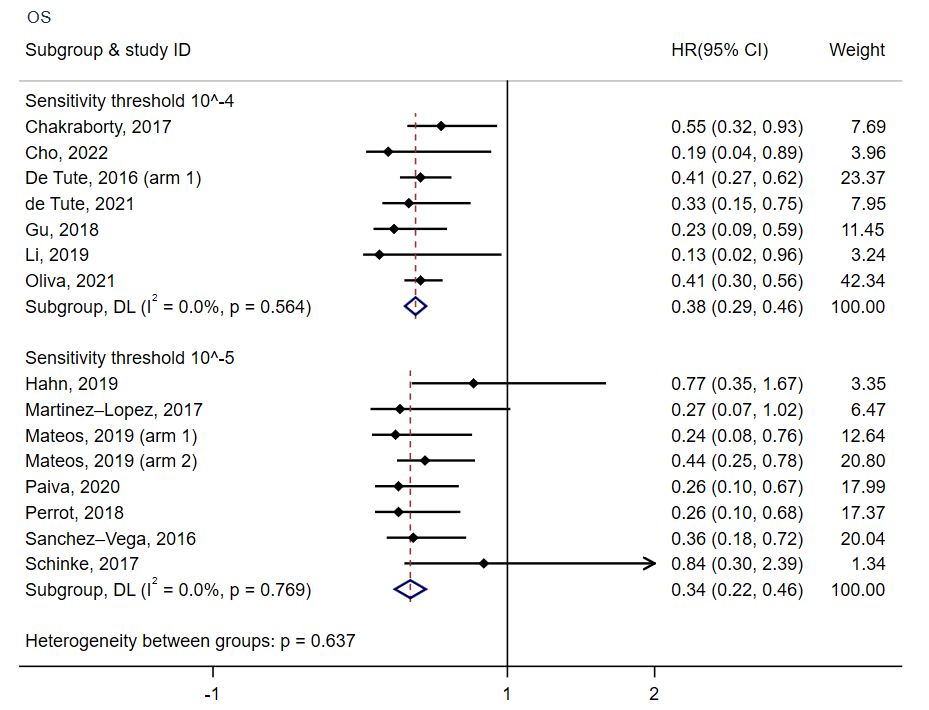


*10^-4^ means 10^-4^ to 10^-5^ sensitivity; 10^-5^ means under 10^-5^ sensitivity

**G)** **Meta-analysis of overall survival according to MRD negativity after ASCT or chemotherapy**


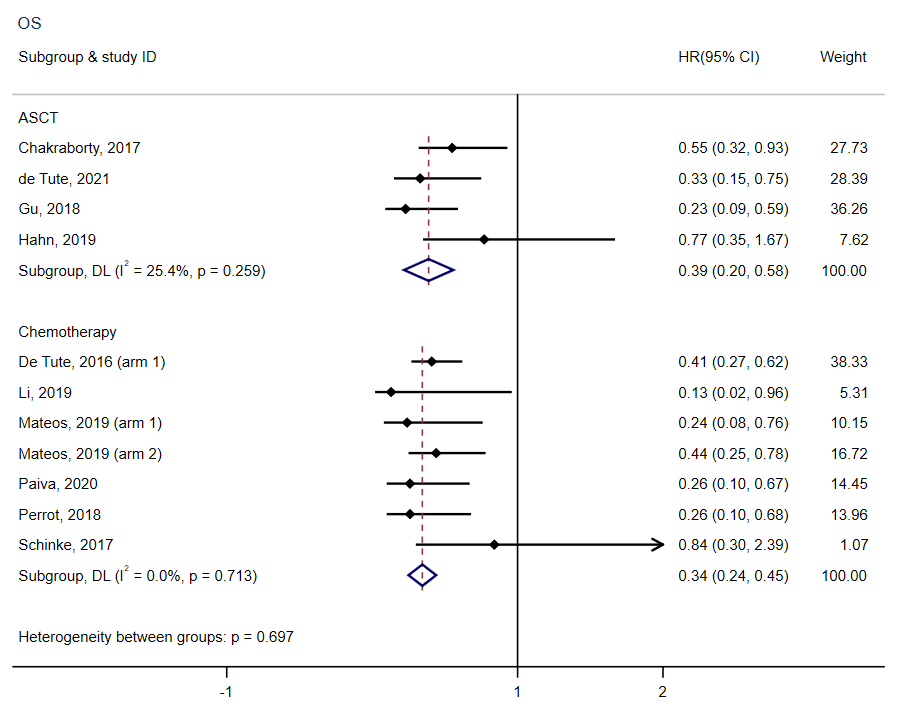


***ASCT,** autologous hematopoietic stem cell transplantation

**H)** **Meta-analysis of overall survival according to MRD negativity at different time points after treatment (under 6 months, 6-12 months, and over 12 months)**


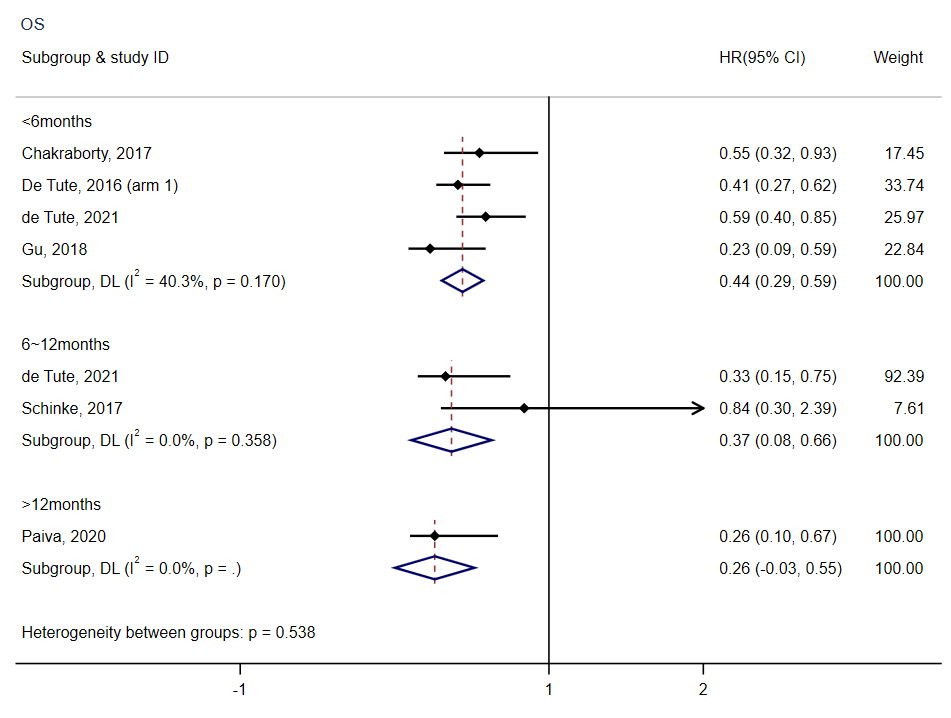


**.**

**Key Question 12. Is it useful to start treatment immediately in patients with high-risk smoldering multiple myeloma?**

**Table 12. Summary of Findings table**

| **Outcomes** | **Illustrative comparative risks***  **(95% CI)** | | **Relative effect (95% CI)** | **No of Participants (studies)** | **Quality of the evidence (GRADE)** | **Comments** |
| --- | --- | --- | --- | --- | --- | --- |
|  | Assumed risk | Corresponding risk |  |  |  |  |
| **PFS** | **558 per 1000** | **224 per 1000** (171 to 284) | **HR 0.31**  (0.23 to 0.41) | 378 (3 studies) | ⊕⊕⊕⊝ **moderate** |  |
| **OS** | **613 per 1000** | **418 per 1000** (276 to 598) | **HR 0.57**  (0.34 to 0.96) | 119 (1 study) | ⊕⊕⊝⊝ **low** |  |
| **AE** | **141 per 1000** | **494 per 1000** (56 to 1000) | **RR 3.51**  (0.40 to 30.45) | 303 (2 studies) | ⊕⊕⊝⊝ **low** |  |
| *The basis for the assumed risk (e.g. the median control group risk across studies) is provided in footnotes. The corresponding risk (and its 95% confidence interval) is based on the assumed risk in the comparison group and the relative effect of the intervention (and its 95% CI). CI, Confidence interval; RR, Risk ratio; HR, Hazard ratio; PFS, progression-free survival; OS, overall survival; AE, adverse event | | | | | | |

**Figure 12. Results of meta-analysis**

**A) Survival**


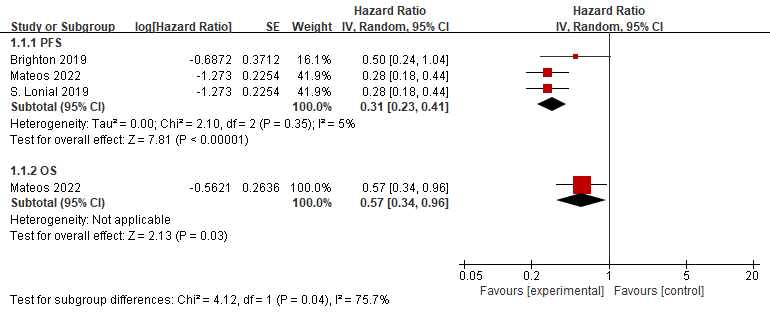


**B) Adverse events**


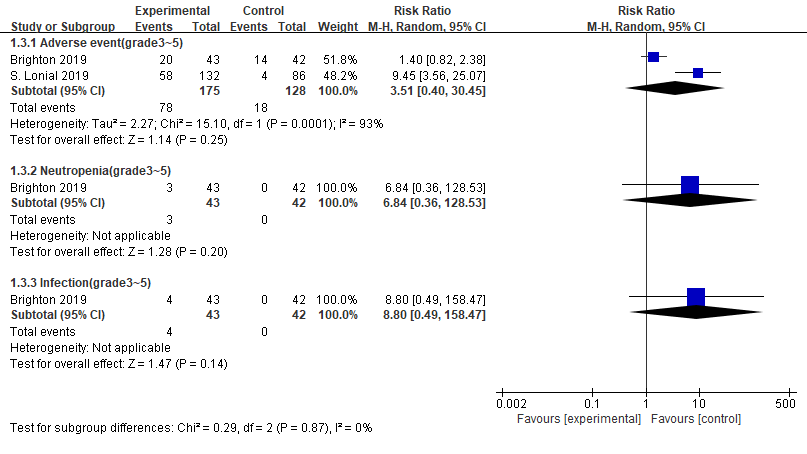

Supplement: Supplementary file 1 — Supplementary Material 1. [file 44313_2025_55_MOESM1_ESM.docx]
